# *Yarrowia lipolytica* as a Platform for Punicic Acid Production

**DOI:** 10.3390/ijms24108823

**Published:** 2023-05-16

**Authors:** Veronika Urbanikova, Young-Kyoung Park, Daniela Krajciova, Mehdi Tachekort, Milan Certik, Ioana Grigoras, Roman Holic, Jean-Marc Nicaud, Peter Gajdos

**Affiliations:** 1 Institute of Biotechnology, Faculty of Chemical and Food Technology, Slovak University of Technology, 81237 Bratislava, Slovakiamilan.certik@stuba.sk (M.C.); 2 Université Paris-Saclay, INRAE, AgroParisTech, Micalis Institute, 78350 Jouy-en-Josas, France; 3 Institute of Animal Biochemistry and Genetics, Centre of Biosciences, Slovak Academy of Sciences, 84005 Bratislava, Slovakiaroman.holic@savba.sk (R.H.); 4 Université Paris-Saclay, Univ Evry, CNRS, CEA, Génomique métabolique, 91057 Evry-Courcouronnes, France; ioana.popescu@univ-evry.fr

**Keywords:** *Yarrowia lipolytica*, punicic acid, synthetic biology, fatty acid conjugase, conjugated fatty acids, conjugated linolenic acid (CLnA), single-cell oil, lipid

## Abstract

Punicic acid (PuA) is a polyunsaturated fatty acid with significant medical, biological, and nutraceutical properties. The primary source of punicic acid is the pomegranate seed oil obtained from fruits of trees that are mainly cultivated in subtropical and tropical climates. To establish sustainable production of PuA, various recombinant microorganisms and plants have been explored as platforms with limited efficiencies. In this study, the oleaginous yeast *Yarrowia lipolytica* was employed as a host for PuA production. First, growth and lipid accumulation of *Y. lipolytica* were evaluated in medium supplemented with pomegranate seed oil, resulting in the accumulation of lipids up to 31.2%, consisting of 22% PuA esterified in the fraction of glycerolipids. In addition, lipid-engineered *Y. lipolytica* strains, transformed with the bifunctional fatty acid conjugase/desaturase from *Punica granatum* (PgFADX), showed the ability to accumulate PuA de novo. PuA was detected in both polar and neutral lipid fractions, especially in phosphatidylcholine and triacylglycerols. Promoter optimization for PgFADX expression resulted in improved accumulation of PuA from 0.9 to 1.8 mg/g of dry cell weight. The best-producing strain expressing PgFADX under the control of a strong erythritol-inducible promoter produced 36.6 mg/L PuA. These results demonstrate that the yeast *Y. lipolytica* is a promising host for PuA production.

## 1. Introduction

Punicic acid [PuA; (9*Z*, 11*E*, 13*Z*)-octadecatrienoic acid] is an ω-5 polyunsaturated fatty acid (FA) with 18 carbons and three conjugated double bonds. PuA is classified as a conjugated linolenic acid (CLnA) [1]. It is highly sought for its potential nutraceutical benefits and significant biological properties such as anti-inflammatory, antilipidemic, antidiabetic, and anticancer activity against several forms of cancer [2,3,4,5,6,7,8], as well as for its preventive role in various neurodegenerative disorders [9]. PuA occurs naturally as a component of plant triacylglycerols (TAGs) [10]. The richest source of this CLnA is pomegranate (*Punica granatum*) seed oil (PSO), which contains up to 83% PuA [11]. Although other plants such as *Trichosanthes kirilowii* and *Momordica balsamina* have been identified as PuA producers, the PuA content in their seed oils is relatively lower, reaching 39% and 51% PuA, respectively [12,13]. PuA is primarily derived from pomegranate seeds. As pomegranate requires a long hot and dry season to thrive, it grows only in the subtropical to tropical climates of the Mediterranean Basin, Asia, America, South Africa, and Australia [14]. With the recent emergence of health benefits associated with PuA, the demand for PSO and pomegranates, in general, has surged over the past decade. To address this demand, there have been efforts to develop a more efficient source of PuA through metabolic engineering of plants and microorganisms.

**Figure 1:**
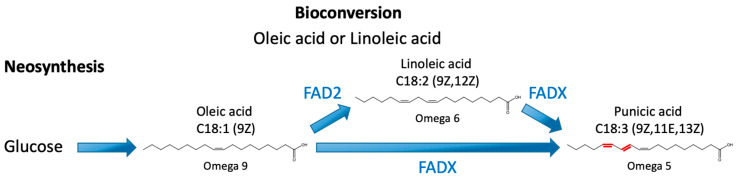
Punicic acid biosynthesis. Punicic acid (PuA) can be synthesized via neosynthesis from phosphatidylcholine and a carbon source such as glucose or via bioconversion using oleic acid or linoleic acid. The oleic moiety can undergo desaturation by a Δ12-desaturase (FAD2) giving rise to linoleic acid, which undergoes another desaturation and isomerization by a specific bifunctional fatty acid conjugase/desaturase (FADX), giving rise to PuA. The enzyme FADX can also directly transform oleic acid into PuA. Note that the process of bioconversion takes place on acyl moiety esterified to molecule of phosphatidylcholine. The introduced double bonds are highlighted in red.

To produce PuA recombinantly, it is necessary to express the bifunctional fatty acid conjugase/desaturase (FADX), which converts the Δ12-double bond of linoleic acid into the Δ11-*trans* and Δ13-*cis* double bonds of PuA (Figure 1). This reaction is catalyzed when the acyl moiety is esterified to a molecule of phosphatidylcholine (PC) [15,16]. FADX has a primary sequence closely related to Δ12-desaturases, and several reports have confirmed its classification as a bifunctional enzyme capable also of converting oleic acid into linoleic acid [16,17,18].

The highest relative PuA content reported to date, 25.1% of total fatty acids (TFAs) (34.33 µg/mL of culture), was achieved in the fission yeast *Schizosaccharomyces pombe* via co-expression of *PgFADX* along with the fatty acid desaturase PgFAD2 [18]. This high level of PuA accumulation in *S. pombe* was achieved using a similar gene combination to that in *Arabidopsis thaliana* [19]. To further improve PuA production in recombinant microorganisms, the nonconventional oleaginous yeast *Yarrowia lipolytica* seems a better choice as it has the ability to accumulate up to 20–40% of lipids in its biomass, and its lipid content can be increased to 90% using metabolic engineering strategies [20,21,22]. As an easily genetically tractable microorganism, *Y. lipolytica* is widely used as a platform for the production of usual and unusual FAs [23]. Examples include odd-chain FAs [24], polyunsaturated FAs such as eicosapentaenoic acid [25,26], α-linolenic acid [27], conjugated FAs [28,29,30], very-long-chain FAs [31], hydroxylated FAs such as ricinoleic acid [32], or cyclopropane FAs [33].

Various *Y. lipolytica* chassis strains have been developed to enhance lipid accumulation and prevent FA degradation. Some recombinant strains of *Y. lipolytica* have been engineered to have either Δ*pox1-6* [34] or Δ*mfe* genotype [35] to abolish the process of β-oxidation. Other recombinant strains of *Y. lipolytica* were designed to be incapable of remobilizing TAGs from lipid bodies. These strains have a TGL lipase deletion (Δ*tgl4* or Δ*tgl3*), which enhances their TAG and overall lipid accumulation capacities [36]. Additionally, the lipid content in yeast cells has been increased through overexpression of glycerol-3-phosphate dehydrogenase GPD1 and diacylglycerol acyltransferase DGA2, two enzymes involved in TAG biosynthesis [37,38]. Thus, a particularly successful *Y. lipolytica* chassis strain, JMY3820, with genotype Δ*pox1-6* Δ*tgl4 pTEF-DGA2 pTEF-GPD1*, was constructed; it is hereafter referred to as the “obese” strain due to its high capacity of lipid overaccumulation [39]. In addition, *Y. lipolytica* can utilize oils through the secretion of extracellular lipases. Studies have identified up to 16 lipase genes in *Y. lipolytica* [40]. Among them, Lip2 is responsible for most of the lipase activity [41], while Lip7 is substrate-specific for longer fatty acid esters (>C9), and Lip8 prefers shorter chains [42].

In this study, we aimed to investigate the potential of the nonconventional yeast *Y. lipolytica* to accumulate and produce PuA. Initially, we tested the ability of this yeast to utilize PSO as a substrate for PuA accumulation by supplementing the culture medium with various concentrations of PSO. Our results show that *Y. lipolytica* is capable of efficient utilization of PSO, releasing PuA from it, and incorporating PuA into various cellular lipids. We then proceeded to explore the capability of *Y. lipolytica* to synthesize and accumulate PuA by overexpressing either the *PgFADX* or the *TkFADX* under the control of either constitutive or inducible promoters to further improve PuA accumulation.

## 2. Results

### 2.1. Lipid and Fatty Acid Profiling of Y. lipolytica Cells Grown in Medium Supplemented with Pomegranate Seed Oil

In our previous studies, we showed that *S. pombe* and *Saccharomyces cerevisiae* can be engineered for PuA production [18,43]; however, these model yeasts are not adapted for high lipid accumulation. In addition, these yeasts are not able to utilize TAG as a substrate due to the lack of extracellular lipase activity. In contrast, *Y. lipolytica* accumulates a high level of lipids and is able to grow in oil-containing media. Therefore, we first investigated the capability of this nonconventional yeast to accumulate PuA by growing in the presence of PSO. Neutral lipid analysis of PSO used for a feeding experiment showed that the PSO consists of TAG structures containing two and three molecules of punicyl-acyls attached to a glycerol backbone, and it contains only a negligible amount of free PuA (Supplementary Figure S1).

Initially, we evaluated that *Y. lipolytica* utilizes PSO and accumulates PuA using different concentrations of PSO (0.01%, 0.05%, 0.1%, and 0.5%) in the presence of 0.5 g/L glucose. The lipid content increased from 11.3% to 31.2% (Supplementary Figure S2a) with a decrease in *Y. lipolytica*’s main FAs, concomitantly with an increase in PuA in the lipid profile (Supplementary Figure S2b). To further confirm the accumulation of TAGs, we examined the structure and morphology of lipid bodies by microscopy. Yeast cells were grown in glucose liquid medium containing PSO. An enlargement of lipid bodies with increasing PSO concentration from 0.1% to 0.5% was observed (Supplementary Figure S3). These results demonstrate that, in those conditions, *Y. lipolytica* was able to hydrolyze PSO and incorporate PuA into intracellular lipids, resulting in an increase in the size of the lipid bodies (Supplementary Figure S3).

**Figure 2:**
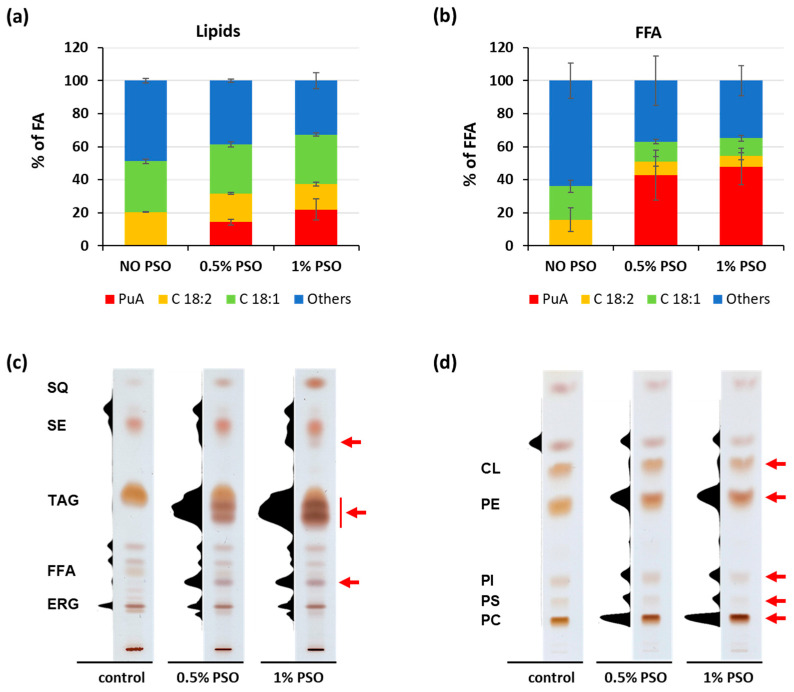
Lipid and fatty acid profiles of the obese *Y. lipolytica* strain JMY3820 grown on YPD supplemented with PSO: (**a**) composition of FA in cellular lipids; (**b**) composition of FFA; (**c**) TLC of neutral lipids, showing densitogram (276 nm) and visualization; (**d**) TLC of polar lipids, showing densitogram (276 nm) and visualization. The red arrow indicates the presence of PuA. Abbreviations: C18:1 (oleic acid), C18:2 (linoleic acid), others (C16:0, C16:1, and C18:0), FA (fatty acids), FFA (free fatty acids), ERG (ergosterol), CL (cardiolipin), PC (phosphatidylcholine), PE (phosphatidylethanolamine), PI (phosphatidylinositol), PL (phospholipid), PS (phosphatidylserine), PSO (pomegranate seed oil), PuA (punicic acid), SQ (squalene), SE (steryl ester), and TAG (triacylglycerol).

Secondly, to evaluate the PuA accumulation capacity of *Y. lipolytica*, determine the PuA-containing lipids, and analyze the effect of PuA on biomass production, feeding with 0.5% and 1.0% PSO was performed, and PuA levels in esterified FAs and in free FAs (FFA) were determined (Figure 2).

The FA analysis by gas chromatography was performed for the characterization and comparison of FA profiles of the esterified FAs and FFAs of *Y. lipolytica* strain grown in medium supplemented with PSO (Figure 2a,b, respectively). PuA reached 14.3% and 21.9% in esterified FAs upon the supplementation of 0.5% and 1% PSO, respectively. The same cells accumulated 42.8% and 47.9% in FFAs, respectively (Supplementary Table S1). This result confirmed that *Y. lipolytica* can accumulate relatively high levels of PuA.

To determine the PuA distribution in lipids after feeding with PSO, neutral lipids and phospholipids were separated by TLC, and the presence of PuA was confirmed on the basis of its conjugated system of double bonds via scanning of the TLC plate at 276 nm. Figure 2c shows the separation of neutral lipids of the *Y. lipolytica* strains by TLC. In the case of neutral lipids, free PuA and PuA incorporated in TAG were identified. Unfortunately, its presence in steryl esters (SEs) could not be determined because of the limitation of the used scanning method. In addition, PuA was effectively incorporated into major phospholipid molecules, namely, PC, phosphatidylethanolamine, phosphatidylinositol, phosphatidylserine, and cardiolipin (Figure 2d). These results confirm that *Y. lipolytica* cell can release PuA from extracellular PSO, internalize free PuA from the medium, activate it with endogenous fatty acyl-CoA synthetases, and incorporate it into various intracellular lipids.

### 2.2. Pomegranate Seed Oil Supplementation Leads to the Accumulation of Lipid Bodies in Y. lipolytica

**Figure 3:**
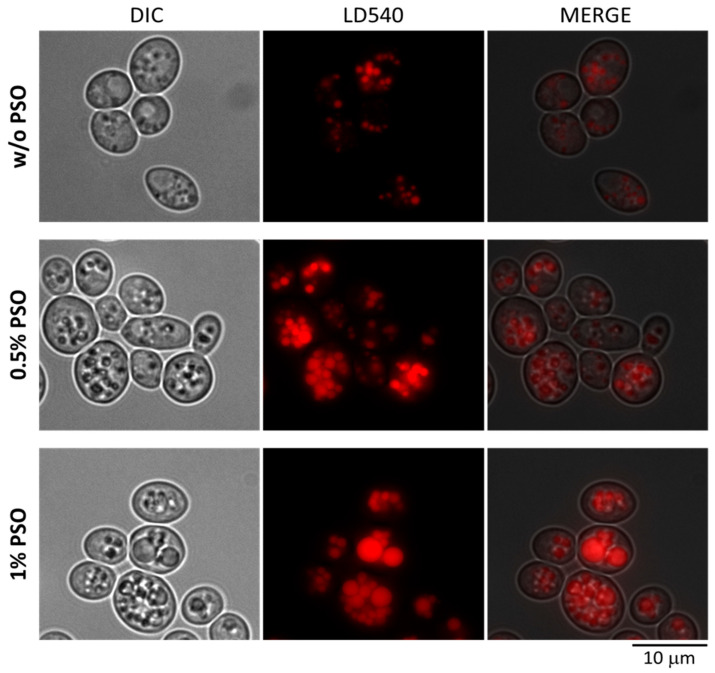
Microscopy of strain JMY3820 grown on YPD supplemented with PSO of indicated concentrations for 72 h. The red color represents the staining of lipid bodies visualized by LD540 a lipid body-specific dye. Images were obtained using a Leica DM5500 fluorescence microscope (Leica Microsystems, Wetzlar, Germany) with a 100× HCXFL OIL objective and filter system Y3 for CY3 green and Leica DFC340 FX Digital Cam for fluorescence microscopy. LAS X Leica software platform (Version 3.0) was used for image acquisition.

Additional microscopy experiments were performed with supplementation of 0.5% and 1% PSO to cells to analyze whether *Y. lipolytica* can further increase the size of its lipid bodies. Lipid bodies were visualized by staining living cells with a lipid body-specific dye LD540. As shown in Figure 3 we observed numerous lipid bodies of bigger size in cells grown with 0.5% PSO compared to control cells. An increase in PSO concentration to 1% was accompanied by a decreased number of even further enlarged lipid bodies. Taken together, our results suggest that *Y. lipolytica* could be a good host for the heterologous production of a single-cell oil containing PuA via metabolic engineering techniques.

### 2.3. Neosynthesis of PuA in Y. lipolytica

Two FADXs, PgFADX from *P. granatum* and TkFADX from *T. kirilowii*, were expressed under control of the constitutive promoter *pTEF* in a *Y. lipolytica* strain engineered to accumulate high amount of lipids JMY3820 [39]. PuA was detected only in the *PgFADX*-expressing strain; therefore, we decided to use this gene for further strain engineering. From the production of PuA in other yeasts (*S. cerevisiae* and *S. pombe*), it is known that PuA production affects the cellular growth [18,43]. In order to separate the production of PuA from the growth, inducible expression of FADX was applied to *Y. lipolytica*. The synthetic erythritol-inducible promoters based on *pEYK1* and *pEYD1* were used in this study. In our previous report, tandem repeats of the upstream activating sequence (*UAS*) of *pEYK1* showed increased expression and induction with the presence of erythritol [44]. The hybrid promoter including *UAS* of *pEYK1* and core *pTEF* also showed a strong expression upon induction. In order to find the optimal promoter for PuA production, we chose three synthetic inducible promoters, *pEYK1 4AB*, *pEYD1*, and *pEYK1 4AB-coreTEF*. The inducible promoters were tested in the *EYK1*-deleted strain JMY8709, which showed a higher expression level compared to the *EYK1*-harboring strain in the previous study [44]. The production of PuA by expressing *FADX* under the control of different promoters was evaluated in modified YPD (6% glucose) or YPDE (6% glucose and 2% erythritol) medium depending on the strain genotype (Table 1). The PuA production via constitutive expression reached 17.3 mg/L with 18.6 g/L dry cell weight (DCW). When the inducible expression was used, the production of PuA was variable depending on the promoters. The *pEYK1 4AB-coreTEF* promoter resulted in the highest production of PuA, i.e., 36.6 mg/L, which is 2.1-fold higher than the expression under *pTEF* constitutive promoter. The expression under *pEYK1 4AB* promoter showed twofold lower production of PuA compared to *pEYK1 4AB-coreTEF*, but still comparable to the production from *pTEF* expression. This result is consistent with our previous reports that showed higher expression by exchanging the core promoter from *pEYK1* to *pTEF* when the reporter protein was used. The expression of *FADX* under *pEYD1* produced only 4.7 mg/L of PuA, resulting in the lowest production in this experiment.

### 2.4. Recombinant Strains Contain PuA in Polar and Neutral Lipids

Metabolic screening of several transformants of the same genotype showed that certain transformants accumulated fewer lipids than corresponding parental strains (Supplementary Table S2). Further investigation revealed that these transformants had lost the *DGA2* overexpression cassette [45] and, thus, lost the obese phenotype (Supplementary Figure S4). This resulted in a higher relative percentage of PuA in transformants without *DGA2* overexpression due to a lower accumulation of major FAs, such as palmitic, palmitoleic, and oleic acids. Therefore, only the results attained in *DGA2*-overexpressing strains are further listed and discussed. Table 2 shows that PuA formed less than 1% of all esterified FAs in selected strains. Its direct precursor linoleic acid formed 2–5% of all esterified FAs. In all strains, the major FA was oleic acid. The relatively low percentage of PuA in lipids was caused by a very strong accumulation of oleic acid. Conversion of oleic acid to linoleic acid was relatively low in *Y. lipolytica*, which also influenced PuA production since linoleic acid is the direct precursor.

**Figure 4:**
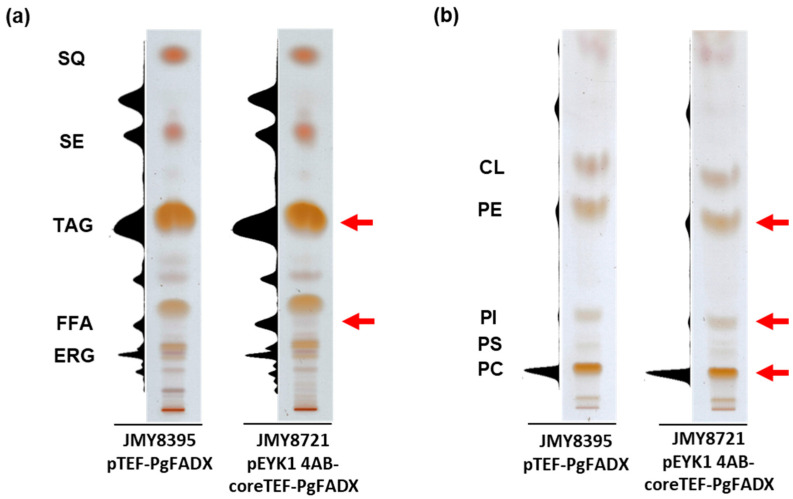
Lipid profile of recombinant *Y. lipolytica* strains grown on YPD medium containing 6% (*w*/*v*) glucose: (**a**) TLC of neutral lipids, showing densitogram (276 nm) and visualization; (**b**) TLC of polar lipids, showing densitogram (276 nm) and visualization. JMY8395 (URA3ex-*pTEF-PgFADX*) and JMY8721 (Δ*eyk1* + URA3ex-*pEYK1 4AB-coreTEF-PgFADX*). The red arrow indicates the presence of PuA. Abbreviations: SQ (squalene), SE (steryl ester), TAG (triacylglycerol), FFA (free fatty acid), ERG (ergosterol), CL (cardiolipin), PE (phosphatidylethanolamine), PI (phosphatidylinositol), PS (phosphatidylserine), and PC (phosphatidylcholine).

Lipid classes of strains JMY8395 and JMY8721 were analyzed by TLC (Figure 4). Compared to the cells of obese strain JMY8320, when PuA was synthesized in recombinant yeast cells, PC was the prominent structure of polar lipids containing PuA. In neutral lipids, PuA was detected in TAG and FFA fractions. There are other lipid classes such as diacylglycerols (DAGs) and SEs which may also contain PuA; however, SEs include sterols, which give a signal at 276 nm, while DAGs are also in close proximity to free sterols, which could have interfered with analysis at 276 nm.

## 3. Discussion

PuA, as the main component of PSO, possesses antidiabetic, anti-obesity, antiproliferative, and anticarcinogenic activities [46], which gives PSO the potential to be used as a nutritional supplement. However, a significant disadvantage of using PSO as a nutraceutical is the fact that pomegranate seeds contain only about 22 g of oil per 100 g of seeds [47,48]. This is an insufficient and unprofitable amount from the point of view of the climatically limited worldwide production of pomegranate. All these aspects are reflected in the relatively high price of pomegranate seed oil (8–50 USD per kilogram, www.alibaba.com, accessed on 5 December 2022). Genetic engineering of suitable organisms could be one of the most promising solutions to this problem. After attempts to produce PuA using transgenic plants [17,19,49,50,51], as well as microorganisms such as *S. cerevisiae* [16,43] and *S. pombe* [18], the *PgFADX* gene responsible for the production of this FA was inserted this time into the genome of oleaginous yeast *Y. lipolytica*. Previously, genes encoding FADX from *P. granatum* (*PgFADX*) and *T. kirilowii* (*TkFADX*) have been expressed in several microbial and plant hosts with the aim of producing PuA (Table 3). The first successful experiment to produce and accumulate PuA was carried out with the model yeast *S. cerevisiae* by expressing *PgFADX* [16]. The *S. cerevisiae* strain was only able to synthesize PuA up to 1.6% (*w*/*w*) of TFA when the growth medium was supplemented with linoleic acid [16]. Recent efforts to improve PuA production in *S. cerevisiae* have resulted in enhanced accumulation levels (3.4% PuA of TFA) through a “push–pull” strategy [43]. This required the use of a strain deleted for the *SNF2*, encoding a transcription factor SNF2 involved in the regulation of lipid accumulation [52], and of co-expression of PgFADX with either phospholipid diacylglycerol acyltransferase (PgPDAT) or lysophosphatidylcholine acyltransferase (PgLPCAT), along with supplementation of the culture medium with 0.05% linoleic acid [43]. The production of PuA was also attempted in the model plant *A. thaliana* by expressing *PgFADX* or *TkFADX*, and the transgenic plants accumulated up to 4.4% (*w*/*w*) and 10.2% (*w*/*w*) of TFA as PuA, respectively [17]. Furthermore, overexpression of PgDGAT2 acyltransferase improved the relative amount of PuA 1.6-fold, reaching 24.8% of TFA, which is the highest level of PuA reported in transgenic plants to date [19].

In this study, before any genetic modification, the ability of *Y. lipolytica* to accumulate PuA and the effect of PuA on the lipid profile and lipid accumulation were tested by cultivation of this yeast in the presence of PSO. In contrast to the model yeasts *S. cerevisiae* and *S. pombe*, the oleaginous yeast *Y. lipolytica* secretes extracellular lipases degrading TAGs to glycerol and FFAs, which are subsequently internalized and further metabolized by the cells [41,42]. This advantage was used in the feeding experiment of *Y. lipolytica* with PSO. The result of the PSO feeding is an observation that *Y. lipolytica* cells can internalize PuA and incorporate it into major phospholipids and storage lipids. More importantly, PuA was found in TAG molecular species containing PuA at all three positions on the glycerol backbone. In addition, the relative content of PuA reached a comparable level as in the recombinant yeast *S. pombe* producing PuA via neosynthesis [18]. Since the oleaginous yeast *Y. lipolytica* is capable of accumulation of much higher levels of lipids, the actual yield of PuA was significantly higher than in the recombinant fission yeast. Fluorescence microscopy showed that the number of lipid bodies and their size increased after supplementation of PSO. Therefore, lipid bodies must have been packed with TAGs containing PuA. To summarize the supplementation experiments, the results showed that the *Y. lipolytica* cell can tolerate a relatively high level of intracellular PuA. This also suggests that obese *Y. lipolytica* cells could be a good platform for heterologous production of PuA via metabolic engineering techniques.

While *Y. lipolytica* has a large potential for ex novo PuA accumulation as was seen in supplementation experiments, de novo production of PuA is much lower compared to the usual FAs of this yeast. This study showed that PuA in yeast cells is synthesized on phospholipids, especially on PC, which is in good agreement with the situation in the pomegranate seeds [15]. It is worth emphasizing that the production of PuA in recombinant strains was influenced by the promoter used for PgFADX expression. The most effective production was achieved with hybrid inducible promoter *pEYK1 4AB-coreTEF*. On the other hand, the expression of PgFADX from promoter *pEYD1* resulted in almost no production of PuA in recombinant *Y. lipolytica*. This is in line with previous study [44], where these native and hybrid promoters were developed. However, the use of a strong promoter and resulting overexpression of PgFADX were not enough to increase PuA production, at least on the level of linoleic acid, the native FA for *Y. lipolytica*. It could be speculated that the key factor for a successful PuA accumulation is the channeling of PuA from phospholipids to TAG. In pomegranate seeds, this is carried out by PDAT directly or by LPCAT via the acyl-CoA pool pathway [43]. In yeast cells, this could be achieved only through the activity of the PDAT enzyme encoded by *LRO1* [54]. Since LRO1 is not as efficient in TAG synthesis as DGA1 or DGA2 [38], the resulting quantity of TAGs containing PuA is rather limited. Taken together, our results showed, despite the use of different strong constitutive or inducible promoters, that PuA synthesis was lower than 1% of TFA.

It is worth emphasizing that de novo lipid accumulation and ex novo lipid accumulation differ in certain aspects. When PSO was supplemented to cells, PuA, together with other FAs of PSO, was released from TAG by extracellular lipases. Since *Y. lipolytica* is a yeast that naturally occupies/colonizes environments containing oils and fats, it is very well equipped with secretory lipases required for extracellular TAG degradation. In addition, it contains a machinery necessary for the efficient import of FFAs released from extracellular TAG. After the transport of external FAs into the cell, FAs are either degraded in peroxisomes or stored in lipid bodies preferentially packed as TAGs. It was previously shown that external FAs are transformed to the corresponding acyl-CoA by YlFAA1 in *Y. lipolytica* and subsequently stored in lipid bodies [55]. Our results indicate that external PuA was also activated by YlFAA1 and became part of the acyl-CoA pool of yeast cells. Since the acyl-CoA pool serves as the source of FAs for all acyltransferases of the Kennedy pathway, punicyl-CoA is accessible also for DGA1 and DGA2, the major TAG-forming acyl-CoA diacylglycerol acyltransferases of *Y. lipolytica*. Therefore, PuA incorporation into TAG and its resulting accumulation were much higher when PSO was supplemented. Similarly, extracellular PuA was transferred to different phospholipids by phospholipid-forming enzymes, while PuA produced de novo in the yeast cells was detected mainly in PC. All this indicates that PuA is the substrate for native acyltransferases of *Y. lipolytica*, but it is important that PuA is present in the acyl-CoA pool, where it can be accessed by various enzymes.

Although the relative amount of PuA in *Y. lipolytica* is low, PuA yield was comparable to other published results in recombinant *S. pombe* cells [18]. We suppose that the reason why oleaginous *Y. lipolytica* did not outcompete non-oleaginous fission yeast is that PuA is not efficiently transferred from phospholipids to storage lipids, represented mainly by TAGs in *Y. lipolytica* cells. While TAG production and overall lipid accumulation in our recombinant *Y. lipolytica* strains were high, a limited presence of PuA could be observed in TAG lipid structures. Therefore, further improvement in the flux of PuA into TAG is needed. This could be achieved by implementing enzymatic activities channeling PuA from PC into an acyl-CoA pool such as LPCAT or through the combined action of phospholipase A2 and long-chain fatty acyl-CoA synthetase (LACS) with enhanced specificities toward PuA. In particular, the latter strategy could be interesting considering a recent observation in recombinant *S. pombe* cells producing calendic acid, a CLnA isomer, where the downregulation of *LCF1*, which encodes the main LACS of fission yeast, was identified [56]. Another explanation for the low accumulation of PuA in TAGs can be related to the ineffective capability of *Y. lipolytica* cells to incorporate PuA into all positions of the glycerol backbone of the TAG molecule when the PuA is produced via neosynthesis. The reason for the observed differences in neosynthesis and PSO feeding is, however, not known.

An alternative approach to establish improved PuA production could be the construction of recombinant *Y. lipolytica* strains secreting FAs into cultivation medium as already performed for ricinoleic acid [57]. Recently, four transporter proteins involved in external FA transport into the cell were discovered [58]. Secretion of PuA into the growth medium via secretion of FA-binding proteins could be another interesting solution, as this type of secretion strategy was demonstrated for squalene, a membrane-impermeable hydrophobic isoprenoid molecule, by recombinant yeast *S. cerevisiae* [59]. In secretion strategies, accumulation of PuA in TAG structures would not be necessary; ultimately, PuA yield could be substantially improved by further optimizations.

Many different approaches have been tested in transgenic plants producing PuA with the aim of increasing PuA content in oil. One particularly interesting strategy was the co-expression of *PgFADX* together with *PgFAD2* and *PgDGAT2* in *A. thaliana* [19]. These engineered plants produced 24.8% PuA in seed oil, which was the highest value for transgenic PuA-producing plants to date. In our study, we employed YlDGA2 which belongs to the DGAT1 family of acyltransferases. Therefore, it could be interesting to also test YlDGA1, a DGAT2 acyltransferase from *Y. lipolytica*, or PgDGAT2 from *P. granatum*.

To conclude, on the basis of these findings, we propose that oleaginous yeast *Y. lipolytica* is a good platform for the production of PuA, and a significant improvement can be obtained via further optimization of its metabolic pathways.

## 4. Materials and Methods

### 4.1. Media Composition and Culture Conditions

*Escherichia coli* strains were grown in lysogeny broth medium (Sigma-Aldrich, Saint-Louis, MO, USA) with the addition of 100 µg/mL ampicillin or 50 µg/mL kanamycin depending on the plasmid, according to a standard protocol [60]. The YNBLeu medium agar plates were used for the selection of yeast transformants. The minimal YNBLeu medium contained 0.17% (*w*/*v*) yeast nitrogen base (without amino acids and ammonium sulfate; BD, Erembodegem, Belgium), 0.5% (*w*/*v*) NH_4_Cl, 50 mM phosphate buffer (pH 6.8), 2% (*w*/*v*) glucose, and leucine (0.1 g/L). Agar plates were prepared via the addition of 2% (*w*/*v*) agar. The yeast inoculum was prepared in rich YPD medium containing 1% (*w*/*v*) yeast extract (BD, Erembodegem, Belgium), 1% (*w*/*v*) peptone (BD, Erembodegem, Belgium), and 2% (*w*/*v*) glucose (Mikrochem, Pezinok, Slovakia).

The modified YPD medium for lipid production contained 1% (*w*/*v*) yeast extract, 2% (*w*/*v*) peptone, and 6% (*w*/*v*) glucose. In the case of strains with the *FADX* gene under the control of an inducible promoter (*pEYK1 4AB*, *pEYD1*, or *pEYK1 4AB-coreTEF*), erythritol was added to the medium at a concentration of 2% (*w*/*v*) (YPDE medium). PSO at different concentrations was added to the medium in the feeding experiment. A 50% PSO suspension was prepared in the presence of 0.14% Tween-40 and sonicated three times for 1 min. PSO (cold-pressed, country of origin: Turkey) was obtained from a local store in Bratislava. the yeast inoculum was prepared in 20 mL of YPD medium in 100 mL flasks and cultivated at 28 °C overnight. Subsequently, the OD-adjusted inoculum (initial OD at 0.1) was inoculated to 50 mL of production medium in 250 mL baffled flasks and cultivated at 28 °C, 130 rpm.

### 4.2. Plasmid and Strain Construction

All strains used in this study are listed in Table 4. *FADX* from *P. granatum* was codon-optimized for *Y. lipolytica* and cloned into the plasmid with different promoters by Golden Gate assembly [61,62]. Plasmids carrying *FADX*-expressing cassettes, JME5213 (GGV-*URA3 ex-pTEF-PgFADX*), JME5215 (GGV-*URA3 ex-pTEF-TkFADX*), JME5334 (GGV-*URA3 ex-pEYK1 4AB-PgFADX*), JME5335 (GGV-*URA3 ex-pEYD1-PgFADX*), and JME5336 (GGV-*URA3 ex-pEYK1 4AB-coreTEF-PgFADX*), were constructed in this study. The complete sequences of all *FADX*-expressing cassettes are mentioned in Supplementary Table S3. Plasmids were digested by NotI before their use in the transformation of *Y. lipolytica* via the lithium acetate method [63]. The transformants were selected on the YNBLeu; then, the positive transformants harboring *FADX*-expressing cassettes were confirmed by colony PCR with the relevant primer pairs (Supplementary Table S4). The PCR amplifications were performed in an Eppendorf 2720 thermal cycler using GoTaq DNA polymerases (Promega, Madison, WI, USA).

### 4.3. Verification of the Presence of the DGA2 Gene

The presence of the DGA2 overexpression cassette was verified by PCR using the pair of primers pTEF-internal-Fw2 and DGA2-Rev. The sequences of these primers are also listed in Supplementary Table S4. In this case, the PCR was carried out in a T100 thermal cycler (Bio-Rad Laboratories, Hercules, CA, USA) in the presence of GoTaq DNA polymerase (Promega, Madison, WI, USA). The length of the amplified fragments was detected by electrophoresis (0.8% agarose in TAE buffer, 90 V, 40 min).

### 4.4. Fluorescence Microscopy Analysis

Lipid droplets in yeast cells were visualized by fluorescence microscopy after staining with LD540 [64,65]. Cells were grown 72 h to stationary phase in YPD medium or YPD medium supplemented with PSO on rotary shaker at 28 °C prior staining. The cell culture was diluted to OD = 2.0; then, the cells were washed three times in 50 mM Tris-HCl, pH 7.5 and suspended in 0.3 mL of 50 mM Tris-HCl, pH 7.5. LD540 (stock solution of 0.5 mg/mL in DMSO) was added to final concentration of 1 μg/mL, and the cells were incubated in the dark for 15 min at room temperature. The stained suspension was washed in 50 mM Tris-HCl (pH 7.5) and suspended in 0.5 mL of 50 mM Tris-HCl (pH 7.5); a 4 μL drop was observed under a fluorescence microscope Leica DM5500 (Leica Microsystems, Wetzlar, Germany) equipped with an Objective HCXFL OIL 100×, filter system Y3 for CY3 green, and Leica DFC340 FX Digital Cam DM5500 (Leica Microsystems, Wetzlar, Germany). Images were processed with LAS software (version 3.0).

### 4.5. Lipid Extraction

Isolation of biomass was performed as follows: cell suspensions were centrifuged (2880× *g*, 5 min), washed twice with saline and 0.5% (*w*/*v*) bovine serum albumin, followed by once with deionized water, and then freeze-dried. The DCW was determined gravimetrically. The total lipid content in the lyophilized biomass was also determined gravimetrically. Next, 45–50 mg of biomass was mixed with 2 mL of methanol, as well as 1 mL of dichloromethane and ballotin, and then continuously shaken for 1.5 h using a Multi Reax shaker (Heidolph, Schwabach, Germany). Afterward, 1 mL of hexane and 1 mL of deionized water were added to this mixture. The mixture was shaken for another 15 min and centrifuged (4668× *g*, 5 min). The organic fraction was collected in a pre-weighed vial. Lipid extraction with hexane was repeated by adding another 1 mL of hexane (shaken for 15 min and centrifuged at 4668× *g* for 5 min). The solvent was completely evaporated using nitrogen gas.

### 4.6. TLC Analysis

An aliquot of lipid extract corresponding to OD10 was applied to silica gel TLC plates (Merck, Darmstadt, Germany) using a semiautomatic sample applicator (CAMAG Linomat 5, Muttenz, Switzerland). Neutral lipids were separated by a two-step TLC solvent system using a method described by Spanova et al. [66] (first step: petroleum ether/diethyl ether/acetic acid, 70:30:2; second step: petroleum ether and diethyl ether, 49:1). Phospholipids were separated by the solvent system (chloroform/methanol/acetic acid/water, 75:45:3:1) as previously reported [67]. The presence of PuA in individual lipids was determined by densitometry at 276 nm (CAMAG TLC Scanner 3, Muttenz, Switzerland). Individual lipid spots were visualized by charring the plates as described previously [68]. Individual peaks were identified using lipid standards.

### 4.7. Fatty Acid Analysis

The FAs present in the isolated oil were converted to the respective FA methyl esters (FAMEs) using a modified method of Glass and Christopherson [69]. Briefly, 100 µL of internal standard (glyceryl tritridecanoate in an amount equivalent to 0.1 mg of C13:0) in hexane and 300 µL of transesterification reagent (0.5 M sodium methoxide with phenolphthalein in methanol) were added to 100 µL of hexane containing approximately 3.6 mg of lipid. The transesterification was carried out for 30 min at room temperature. Subsequently, 150 µL of anhydrous methanolic HCl solution was added. After vortexing, the reaction mixture was decolored. FAMEs were extracted twice with 500 µL of hexane (10 min extraction and centrifugation; 2990× *g*, 5 min) and analyzed by GC-8890 (Agilent Technologies, Santa Clara, CA, USA) as described previously [70]. FFAs were further analyzed using the method described by Garaiova et al. [56].

### 4.8. Data Analysis

Statistical analysis was performed using the software Microsoft Excel (Microsoft 365 version 2023 software pack) equipped with the data analysis tool. Obtained data were processed using single-factor analysis of variance (ANOVA).

## Figures and Tables

**Table 1:** Cultivation of the recombinant *DGA2*-overexpressing strains for 72 h on YPD medium containing 6% (*w*/*v*) glucose for JMY8381 (control strain) and JMY8395 (*URA3ex-pTEF-PgFADX*), and on YPDE medium containing 6% (*w*/*v*) glucose and 2% (*w*/*v*) erythritol for JMY8710 (Δ*eyk1* control strain), JMY8716 (Δ*eyk1* + *URA3ex-pEYK1 4AB-PgFADX*), JMY8719 (Δ*eyk1* + *URA3ex-pEYD1-PgFADX*), and JMY8721 (Δ*eyk1* + *URA3ex-pEYK1 4AB-coreTEF-PgFADX*). For each data point, we used three biological replicates and calculated average and standard deviation values. Statistical analysis of the results confirmed that the mean values differed significantly (*p*-value < 10^−5^).

Strain	*PgFADX* Promoter	DCW (g/L)	TFA/DCW (%)	PuA (µg/mg DCW)	PuA (mg/L)
JMY8381 obese	-	19.1 ± 0.1	34.2 ± 1.9	-	-
JMY8395 obese + *PgFADX*	*pTEF*	18.6 ± 0.6	33.5 ± 1.4	0.9 ± 0.0	17.3 ± 1.3
JMY8710 obese + Δ*eyk1*	-	14.4 ± 0.3	25.5 ± 1.4	-	-
JMY8716 obese+ Δ*eyk1* + *PgFADX*	*pEYK1 4AB*	18.3 ± 0.3	30.9 ± 3.6	1.0 ± 0.1	18.9 ± 1.9
JMY8719 obese + Δ*eyk1* + *PgFADX*	*pEYD1*	19.2 ± 0.6	32.1 ±1.4	0.2 ± 0.1	4.7 ± 1.9
JMY8721 obese + Δ*eyk1* + *PgFADX*	*pEYK1 4AB-coreTEF*	19.9 ± 0.3	31.6 ± 0.4	1.8 ± 0.1	36.6 ± 2.4

**Table 2:** Profile of total esterified fatty acids in recombinant yeast strains. JMY8381 (control strain), JMY8395 (*URA3ex-pTEF-PgFADX*), JMY8710 (Δ*eyk1* control strain), JMY8716 (Δ*eyk1* + *URA3ex-pEYK1 4AB-PgFADX*), JMY8717 (Δ*eyk1* + *URA3ex-pEYD1-PgFADX*), and JMY8721 (Δ*eyk1* + *URA3ex-pEYK1 4AB-coreTEF-PgFADX*). For each data point, we used three biological replicates and calculated average and standard deviation values. Statistical analysis of the results confirmed that the mean values differed significantly (*p*-value < 10^−5^).

Strain	JMY8381	JMY8395	JMY8710	JMY8716	JMY8719	JMY8721
	Obese	Obese + *PgFADX*	Obese + Δ*eyk1*	Obese + Δ*eyk1* + *PgFADX*	Obese + Δ*eyk1* + *PgFADX*	Obese + Δ*eyk1* + *PgFADX*
Promoter		*pTEF*		*pEYK1 4AB*	*pEYD1*	*pEYK1 4AB-coreTEF*
	% of esterified fatty acids					
PuA	-	0.4 ± 0.2	-	0.3 ± 0.0	0.1 ± 0.0	0.5 ± 0.1
C18:2	2.9 ± 0.1	2.7 ± 0.3	5.1 ± 0.1	2.4 ± 0.1	2.6 ± 0.1	2.9 ± 1.0
C18:1	43.9 ± 0.6	50.4 ± 2.7	42.1 ± 0.2	46.9 ± 0.6	44.9 ± 1.5	46.9 ± 4.8
C18:0	11.9 ± 1.1	9.3 ± 1.6	9.0 ± 0.2	12.4 ± 0.1	10.4.0 ± 1.0	11.3 ± 2.0
C16:1	5.6 ± 0.2	6.1 ± 0.2	7.4 ± 0.1	5.3 ± 0.0	5.8 ± 0.2	5.3 ± 0.5
C16:0	30.2 ± 0.6	25.9 ± 1.2	31.7 ± 0.2	27.4 ± 0.5	31.5 ± 0.4	27.6 ± 1.2
others	5.6 ± 0.5	5.3 ± 0.7	4.7 ± 0.2	5.3 ± 0.2	4.7 ± 0.4	5.5 ± 1.4

**Table 3:** Chassis strains for PuA production.

Host	Carbon Source	Mechanism	Promoter	Gene	Fatty Acid Produced	PuA Level (% TFA)	PuA Yield (mg/L)	References
*S. cerevisiae*	Glucose + galactose	Neosynthesis	*pGAL1*	*PgFADX (PuFADX)*	C18:2	n.d.	ND	[16]
*S. cerevisiae*	Glucose + galactose + C18:2	Bioconversion	*pGAL1*	*PgFADX (PuFADX)*	PuA	1.6	ND	[16]
*S. cerevisiae*	Galactose + C18:2	Bioconversion	*pGAL10*	*TkFADX (TkFac)*	C18:2, PuA	0.1	ND	[17]
*S. cerevisiae*	Galactose + C18:2	Bioconversion	*pGAL10*	*PgFADX (PgFac)*	C18:2, PuA	0.8	ND	[17]
*S. cerevisiae* Δ*snf2*	Galactose + C18:2	Bioconversion	*pGAL10*	*PgFADX*, *PgPDAT*, *PgPDCT*/*PgLPCAT*	PuA	3.4	7.2	[43]
*S. pombe*	Glucose	Neosynthesis	*pNMT1*	*PgFADX*	C18:2, PuA	19.6	38.7	[18]
*S. pombe*	Glucose	Neosynthesis	*pNMT1*	*PgFADX*, *PgFAD2*	C18:2, PuA	25.1	34.3	[18]
*A. thaliana*		Neosynthesis	*CaMV*	*TkFADX (TkFac)*	PuA	~0.4		[17]
*A. thaliana*		Neosynthesis	*CaMV*	*PgFADX (PgFac)*	PuA	~0.4		[17]
*A. thaliana*		Neosynthesis	*napin*	*TkFADX (TkFac)*	PuA	10.2		[17]
*A. thaliana*		Neosynthesis	*napin*	*PgFADX (PgFac)*	PuA	4.4		[17]
*A. thaliana fad3 fae1*		Neosynthesis	*napin*	*PgFADX*	PuA	11.5		[53]
*A. thaliana fad3 fae1*		Neosynthesis	*napin*	*PgFADX*, *PgFAD2*	PuA	21.2		[53]
*A. thaliana fad3 fae1*		Neosynthesis	*napin*	*PgFADX*, *PgFAD2*, *PgDGAT2*	PuA	24.8		[19]
*A. thaliana fad3 fae1*		Neosynthesis	*napin*	*PgFADX*	PuA	9.2		[49]
*A. thaliana fad3 fae1*		Neosynthesis	*phaseolin*	*PgFADX*	PuA	9.1		[49]
*A. thaliana fad3 fae1*		Neosynthesis	*linin*	*PgFADX*	PuA	13.2		[49]
*A. thaliana fad3 fae1*		Neosynthesis	*conlinin*	*PgFADX*	PuA	10.3		[49]
*Brassica napus*		Neosynthesis	*napin*	*TkFADX (TkFac)*	PuA	2.5		[50]
*B. napus*		Neosynthesis	*napin*	*PgFADX*, *PgFAD2*	PuA	11.1		[51]

n.d.—not detected; ND—not determined; *napin*, *linin*, *phaseolin*, and *conlinin*—seed-specific promoters; *CaMV*—constitutive 35S promoter; *pGAL1* and *pGAL10*—strong promoters induced by galactose; *pNMT1*—strong inducible promoter repressed by thiamine.

**Table 4:** Strains used in the study.

Strain	Genotype	Reference
***E. coli.***		
JME5213	GGV-*URA3 ex-pTEF-PgFADX*	This study
JME5215	*GGV-URA3 ex-pTEF-TkFADX*	This study
JME5334	GGV-*URA3 ex*-*pEYK1 4AB-PgFADX*	This study
JME5335	GGV-*URA3 ex*-*pEYD1-PgFADX*	This study
JME5336	GGV-*URA3 ex-pEYK1 4AB-coreTEF-PgFADX*	This study
***Y. lipolytica***		
JMY3820	MATa *ura3-302 leu2-270 xpr2-322* Δ*pox1-6* Δ*tgl4* + *pTEF*-*DGA2* + *pTEF-GPD1*	[39]
JMY8381	JMY3820 + *URA3*	This study
JMY8393 JMY8394 JMY8395	JMY3820 + *URA3 ex-pTEF-PgFADX*	This study
JMY8385	JMY3820 + *URA3 ex-pTEF-TkFADX*	This study
JMY8709	JMY3820 Δ*eyk1*	This study
JMY8710	JMY3820 Δ*eyk1* + *URA3*	This study
JMY8714 JMY8715 JMY8716	JMY3820 Δ*eyk1* + *URA3 ex-Peyk1 4AB-PgFADX*	This study
JMY8717 JMY8718 JMY8719	JMY3820 Δ*eyk1* + *URA3 ex-pEYD1-PgFADX*	This study
JMY8720 JMY8721 JMY8722	JMY3820 Δ*eyk1* + *URA3 ex-pEYK1 4AB-coreTEF-PgFADX*	This study

## Data Availability

The data are contained within the article and Supplementary Materials.

## Supplementary Materials

The following supporting information can be downloaded at https://www.mdpi.com/article/10.3390/ijms24108823/s1.
