# Supplementary material for: Yarrowia lipolytica as a Platform for Punicic Acid Production
Source: Int J Mol Sci. 2023 May 16;24(10):8823. doi: 10.3390/ijms24108823 (PMC10219088; doi:10.3390/ijms24108823)
Supplement: Supplementary file 1 [file ijms-24-08823-s001.zip › ijms-2367383-supplementary.pdf]

Supplementary information for:

Yarrowia lipolytica as a platform for punicic acid production

Veronika Urbanikova [1,†], Young-Kyoung Park [2,†], Daniela Krajciova [3], Mehdi Tachekort [2,4], Milan Certik [1], Ioana Grigoras [4], Roman Holic [3,#], Jean-Marc Nicaud [2,#,*] and Peter Gajdos [1,#,*]

1 Faculty of Chemical and Food Technology, Institute of Biotechnology, Slovak University of Technology, Bratislava, Slovakia; milan.certik@stuba.sk
2 Universite Paris-Saclay, INRAE, AgroParisTech, Micalis Institute, 78350, Jouy-en-Josas, France; jean-marc.nicaud@inrae.fr
3 Institute of Animal Biochemistry and Genetics, Centre of Biosciences, Slovak Academy of Sciences, 84005, Bratislava, Slovakia; roman.holic@savba.sk
4 Universite Paris-Saclay, Univ Evry, CNRS, CEA, Genomique Metabolique, Evry, France; ioana.popescu@univ-evry.fr
* Correspondence: peter.gajdos@stuba.sk (P.G.); jean-marc.nicaud@inrae.fr (J.M.N.)
# Authors have jointly supervised the work
† These authors contributed equally to this work.

Table of contents **Page**

Supplementary Figure S1. Neutral lipid profile of PSO. 2

Supplementary Figure S2. Lipid content and fatty acid profiles of *Y. lipolytica* JMY3820 strain upon pomegranate seed oil (PSO) feeding. 3

Supplementary Figure S3. Microscopic imaging of *Y. lipolytica* JMY3820 strain upon pomegranate seed oil (PSO) feeding. 4

Supplementary Figure S4. Verification of the presence of *DGA2* gene in the genome of JMY8394, JMY8395, JMY8720 and JMY8721 strains. 5

Supplementary Table S1. Relative level of PuA in *Y. lipolytica* cells grown in media supplemented with PSO. 6

Supplementary Table S2. Cultivation of all constructed *PgFADX*-expressing *Y. lipolytica* strains. 7

Supplementary Table S3. Sequences of *FADX*-expressing cassettes used in this study. 8

Supplementary Table S4. Primers used in the study. 13

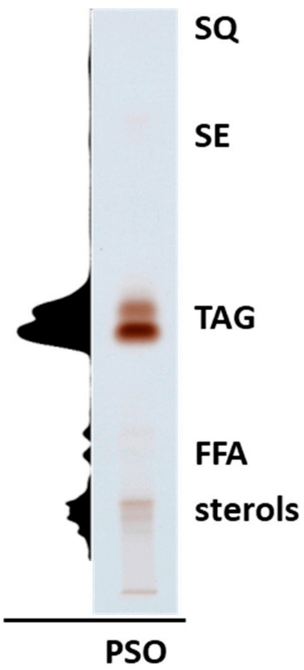

Supplementary Figure S1. Neutral lipid profile of PSO (densitogram (276 nm) and visualization). Abbreviation: SQ (squalene), SE (steryl ester), TAG (triacylglycerol), FFA (free fatty acid).

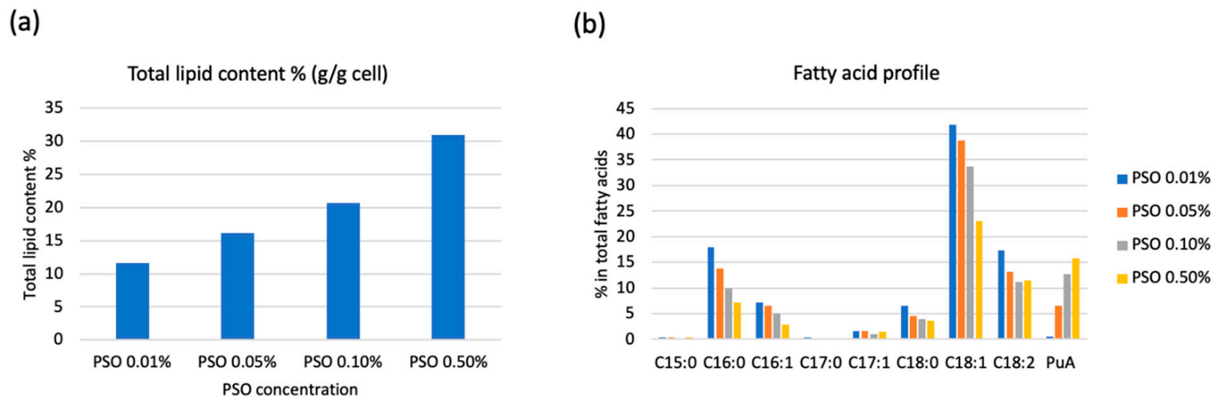

Supplementary Figure S2. Lipid content and fatty acid profiles of *Y. lipolytica* JMY3820 obese strain upon pomegranate seed oil (PSO) feeding. Yeast cells were grown for 72 hours in YPD (2% glucose) media supplemented with four different PSO concentrations (0.01%, 0.05%, 0.1% and 0.5%). (**a**) lipid content as gWW/ gDCW, (**b**) lipid profiles as a percentage of total lipid.

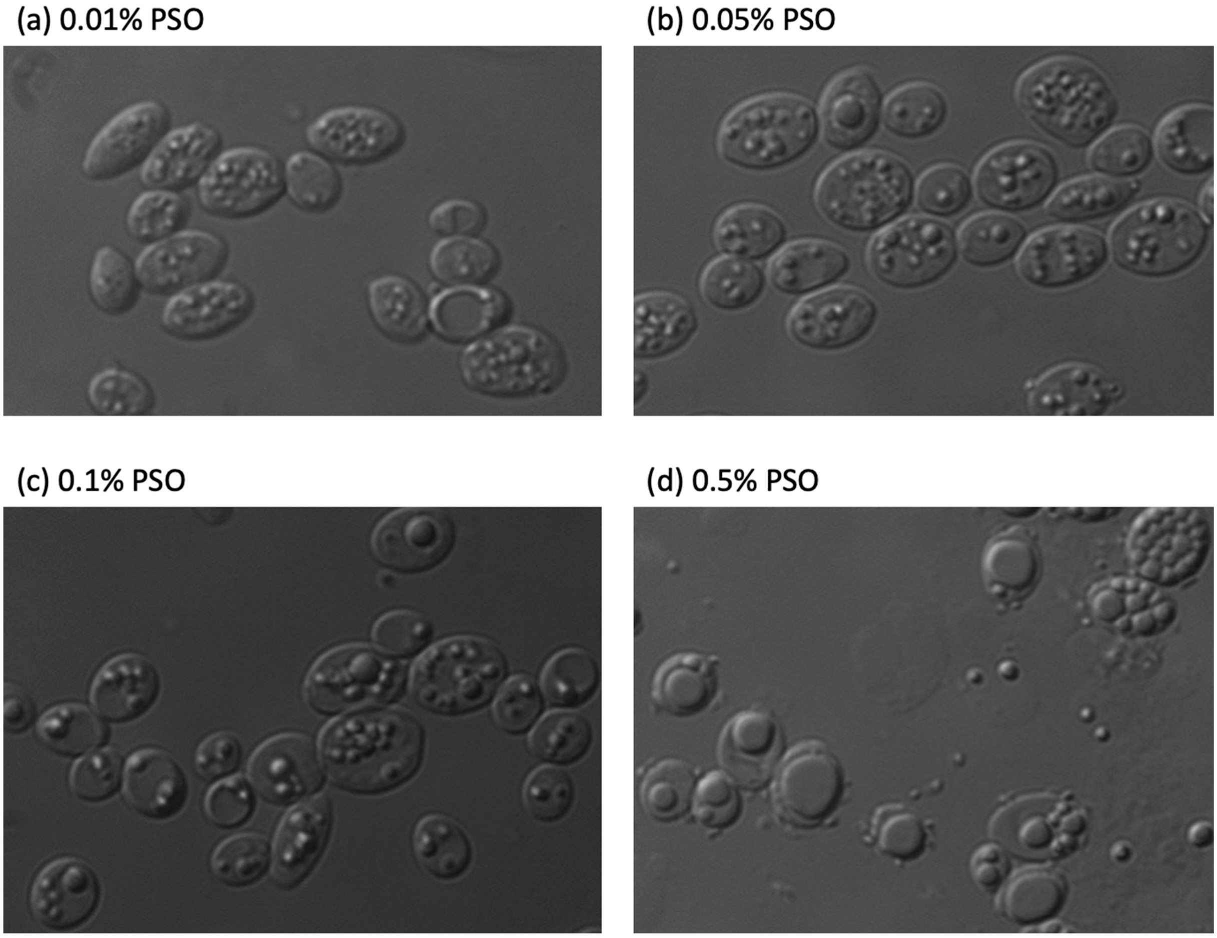

Supplementary Figure S3. Microscopic imaging of *Y. lipolytica* JMY3820 obese strain upon pomegranate seed oil (PSO) feeding. Yeast cells were grown for 72 hours in YPD (2% glucose) media supplemented with four different PSO concentrations: (**a**) 0.01%, (**b**) 0.05%, (**c**) 0.1%, (**d**) 0.5%. Images were obtained using a Zeiss Axio Imager M2 microscope (Zeiss, Le Pecq, France) with a 100× objective lens and Zeiss filter sets 45 and 46 for fluorescence microscopy. Axiovision 4.8 software (Zeiss, Le Pecq, France) was used for image acquisition.

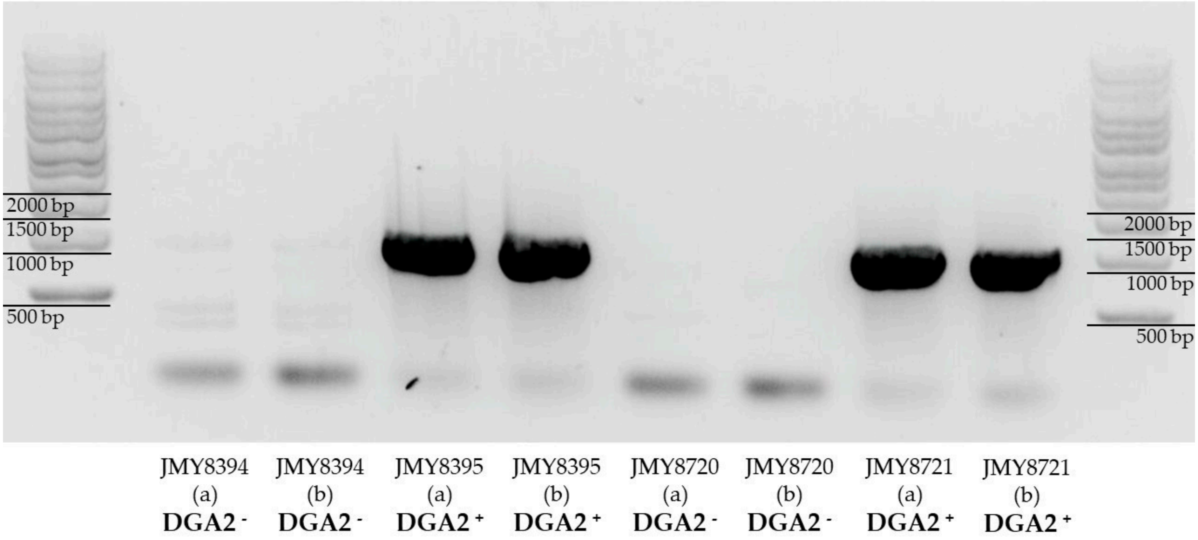

Supplementary Figure S4. Verification of the presence of *pTEF-DGA2* cassette in the genome of JMY8394, JMY8395, JMY8720 and JMY8721 strains. The presence of *pTEF-DGA2* was confirmed by PCR with the pTEF-internal-Fw2 (5′-CCAATTGACCCCAAATTGACC-3′) and DGA2-Rev (5′-ATGAGGCGAGTTTGAGAGACAG-3′) primers and subsequent electrophoresis. The expected length of the fragment was 992 base pairs (bp). Two parallel experiments were performed for each strain.

Supplementary Table S1. Relative level of PuA in *Y. lipolytica* cells grown in media supplemented with PSO.

PSO concentration	PuA in lipids (%)	PuA in FFA (%)
0.5%	14.3 ± 1.7	42.8 ± 15.2
1.0%	21.9 ± 6.3	47.9 ± 11.1

results from three independent experiments

Supplementary Table S2. Cultivation of all constructed *PgFADX*-expressing *Y. lipolytica* strains for 72 hours on YPD medium containing 6% (w/v) glucose for JMY8381 (control strain), JMY8393 (*URA3ex-pTEF-PgFADX*), JMY8394 (*URA3ex-pTEF-PgFADX*), JMY8395 (*URA3ex-pTEF-PgFADX*), and on YPDE medium containing 6% (w/v) glucose and 2% (w/v) erythritol for JMY8710 (*Δeyk1* control strain), JMY8714 (*Δeyk1* + *URA3ex-pEYK1 4AB-PgFADX*), JMY8715 (*Δeyk1* + *URA3ex-pEYK1 4AB-PgFADX*), JMY8716 (*Δeyk1* + *URA3ex-pEYK1 4AB-PgFADX*), JMY8717 (*Δeyk1* + *URA3ex-pEYD1-PgFADX*), JMY8718 (*Δeyk1* + *URA3ex-pEYD1-PgFADX*), JMY8719 (*Δeyk1* + *URA3ex-pEYD1-PgFADX*), JMY8720 (*Δeyk1* + *URA3ex-pEYK1 4AB-coreTEF-PgFADX*), JMY8721 (*Δeyk1* + *URA3ex-pEYK1 4AB-coreTEF-PgFADX*) and JMY8722 (*Δeyk1* + *URA3ex-pEYK1 4AB-coreTEF-PgFADX*).

	PgFADX **promoter**	**DCW (g/L)**	**TFA/DCW (%)**	**PuA (% of esterified FA)**	**PuA (µg/mg DCW)**
**JMY8381 obese**	-	19.1 ± 0.1	34.2 ± 1.9	-	-
**JMY8393 obese^x + *PgFADX***	*pTEF*	15.4 ± 0.8	10.5 ± 0.8	1.6 ± 0.3	1.8 ± 0.4
**JMY8394 obese^x + *PgFADX***	*pTEF*	13.5 ± 0.3	9.2 ± 0.2	1.6 ± 0.0	1.5 ± 0.0
**JMY8395 obese + *PgFADX***	*pTEF*	18.6 ± 0.6	33.5 ± 1.4	0.4 ± 0.2	0.9 ± 0.0
**JMY8710 obese + *Δeyk1***	-	14.4 ± 0.3	25.5 ±1.4		-
**JMY8714 obese + *Δeyk1* + *PgFADX***	*pEYK1 4AB*	14.7 ± 0.2	27.9 ± 4	0.3 ± 0.0	0.9 ± 0.4
**JMY8715 obese + *Δeyk1* + *PgFADX***	*pEYK1 4AB*	17.5 ± 0.9	33.7 ± 0.1	0.3 ± 0.0	1.0 ± 0.1
**JMY8716 obese + *Δeyk1* + *PgFADX***	*pEYK1 4AB*	18.3 ± 0.3	30.9 ± 3.6	0.3 ± 0.0	1.0 ± 0.1
**JMY8717 obese + *Δeyk1* + *PgFADX***	*pEYD1*	17.2 ± 0.6	26.9 ± 1.5	0.02 ± 0.0	0.1 ± 0.0
**JMY8718 obese^x + *Δeyk1* + *PgFADX***	*pEYD1*	12.4 ± 1.0	6.9 ± 0.6	0.4 ± 0.2	0.3 ± 0.2
**JMY8719 obese + *Δeyk1* + *PgFADX***	*pEYD1*	19.2 ± 0.6	32.1 ± 1.4	0.1 ± 0.0	0.2 ± 0.1
**JMY8720 obese^x +*Δeyk1* + *PgFADX***	*pEYK1 4AB-coreTEF*	13.7 ± 0.1	8.8 ± 0.7	3.1 ± 0.2	2.8 ± 0.3
**JMY8721 obese +*Δeyk1* + *PgFADX***	*pEYK1 4AB-coreTEF*	19.9 ± 0.3	31.6 ± 0.4	0.5 ± 0.1	1.8 ± 0.1
**JMY8722 obese^x + *Δeyk1* + *PgFADX***	*pEYK1 4AB-coreTEF*	13.5 ± 0.6	8.0 ± 0.4	2.8 ± 1.0	2.3 ± 0.9

For each data point, we used three biological replicates and calculated average and standard deviation values.
The "x" designation next to the word obese means that the strain has lost the *pTEF-DGA2* overexpression cassette.

Supplementary Table S3. Sequences of *FADX*-expressing cassettes used in this study: the *Not*I fragments used to transform *Y. lipolytica* comprising the PgFADX and TkFADX expression cassettes under the control of different promoters (*pTEF*, *pEYK1-4AB*, *pEYD1AB* or *pEYK1-4AB-coreTEF*) and the *LIP2* terminator, along with an expression cassette for the *URA3* auxotrophic selection marker (*URA3ex*), flanked by two *Zeta* sequences (*ZetaUp* and *ZetaDown*) that allow random integrations into *Y. lipolytica* genome.

FADX gene	*PgFADX*
Promoter	*pTEF*
E. coli strain	JME5213
Y. lipolytica strain	JMY8395

GCGGCCGCTGTCGGGAACCGCGTTCAGGTGGAACAGGACCACCTCCCTTGCACTTCTTGGTATATCAGTATAGGCTG
ATGTATTCATAGTGGGGTTTTTCATAATAAATTTACTAACGGCAGGCAACATTCACTCGGCTTAAACGCAAAACGGA
CCGTCTTGATATCTTCTGACGCATTGACCACCGAGAAATAGTGTTAGTTACCGGGTGAGTTATTGTTCTTCTACACAG
GCGACGCCCATCGTCTAGAGTTGATGTACTAACTCAGATTTCACTACCTACCCTATCCCTGGTACGCACAAAGCACT
TTGCTAGAAGGTATAACTTCGTATAGCATACATTATACGAAGTTATTCTGAATTCCGAGAAACACAACAACATGCCC
CATTGGACAGACCATGCGGATACACAGGTTGTGCAGTACCATACATACTCGATCAGACAGGTCGTCTGACCATCAT
ACAAGCTGAACAGCGCTCCATACTTGCACGCTCTCTATATACACAGTTAAATTACATATCCATAGTCTAACCTCTAA
CAGTTAATCTTCTGGTAAGCCTCCCAGCCAGCCTTCTGGTATCGCTTGGCCTCCTCAATAGGATCTCGGTTCTGGCCG
TACAGACCTCGGCCGACAATTATGATATCCGTTCCGGTAGACATGACATCCTCAACAGTTCGGTACTGCTGTCCGAG
AGCGTCTCCCTTGTCGTCAAGACCCACCCCGGGGGTCAGAATAAGCCAGTCCTCAGAGTCGCCCTTAGGTCGGTTCT
GGGCAATGAAGCCAACCACAAACTCGGGGTCGGATCGGGCAAGCTCAATGGTCTGCTTGGAGTACTCGCCAGTGGC
CAGAGAGCCCTTGCAAGACAGCTCGGCCAGCATGAGCAGACCTCTGGCCAGCTTCTCGTTGGGAGAGGGGACTAG
GAACTCCTTGTACTGGGAGTTCTCGTAGTCAGAGACGTCCTCCTTCTTCTGTTCAGAGACAGTTTCCTCGGCACCAGC
TCGCAGGCCAGCAATGATTCCGGTTCCGGGTACACCGTGGGCGTTGGTGATATCGGACCACTCGGCGATTCGGTGAC
ACCGGTACTGGTGCTTGACAGTGTTGCCAATATCTGCGAACTTTCTGTCCTCGAACAGGAAGAAACCGTGCTTAAGA
GCAAGTTCCTTGAGGGGGAGCACAGTGCCGGCGTAGGTGAAGTCGTCAATGATGTCGATATGGGTCTTGATCATGC
ACACATAAGGTCCGACCTTATCGGCAAGCTCAATGAGCTCCTTGGTGGTGGTAACATCCAGAGAAGCACACAGGTT
GGTTTTCTTGGCTGCCACGAGCTTGAGCACTCGAGCGGCAAAGGCGGACTTGTGGACGTTAGCTCGAGCTTCGTAGG
AGGGCATTTTGGTGGTGAAGAGGAGACTGAAATAAATTTAGTCTGCAGAACTTTTTATCGGAACCTTATCTGGGGCA
GTGAAGTATATGTTATGGTAATAGTTACGAGTTAGTTGAACTTATAGATAGACTGGACTATACGGCTATCGGTCCAA
ATTAGAAAGAACGTCAATGGCTCTCTGGGCGCGAATTCGTATAACTTCGTATAGCAGGAGTTATCCGAAGCGAACG
GGGGTTGGCGGCGTATTTGTGTCCCAAAAAACAGCCCCAATTGCCCCAATTGACCCCAAATTGACCCAGTAGCGGG
CCCAACCCCGGCGAGAGCCCCCTTCACCCCACATATCAAACCTCCCCCGGTTCCCACACTTGCCGTTAAGGGCGTA
GGGTACTGCAGTCTGGAATCTACGCTTGTTCAGACTTTGTACTAGTTTCTTTGTCTGGCCATCCGGGTAACCCATGCC
GGACGCAAAATAGACTACTGAAAATTTTTTTGCTTTGTGGTTGGGACTTTAGCCAAGGGTATAAAAGACCACCGTCC
CCGAATTACCTTTCCTCTTCTTTTCTCTCTCTCCTTGTCAACTCACACCCGAAGAATGGGCGCAGACGGTACTATGAG
CCCCGTGCTTACGAAGCGACGACCTGATCAGGAAATTAATAAGCTCGATATTAAACCCAACCACGAGGTCGATATT
GCACGACGAGCACCCCACTCTAAGCCCCCTTTTACTCTCAGCGATCTTCGTTCTGCTATTCCTCCCCACTGTTTCCAC
CGTTCCCTCCTTATGTCCTCCAGCTACCTTATCCGGGATTTTGCTCTTGCATTTCTTTTCTACCATTCCGCTGTGACCTA
TATCCCCCTCCTTCCTAAACCTCTCGCCTGTATGGCTTGGCCCGTGTACTGGTTTCTCCAGGGATCTAATATGCTCGG
AATTTGGGTTATCGCCCACGAATGCGGACATCAGGCCTTTAGCAATTACGGTTGGGTCAACGACGCAGTCGGTTTCT
TTCTGCATACCAGCCTCCTTGTGCCTTATTTCCCTTTCAAATACTCGCACAGACGTCACCACTCCAACACTAATAGCG
TTGAGCACGATGAGGTTTTTGTTCCTAGACACAAGGACGGCGTCCAATGGTATTATCGGTTCTTCAACAATACGCCT
GGCCGTGTCCTCACACTGACTCTTACACTCCTGGTTGGATGGCCTTCCTACCTTGCTTTTAACGCTTCCGGTAGACCTT
ATGACGGTTTCGCCTCGCATTACAATCCTAATGCCCAAATTTTTAACCTCCGTGAGCGTTTCTGGGTGCACGTGTCGA
ACATCGGCATTCTTGCTATCTACTATATCCTGTACCGACTCGCCACTACGAAGGGCCTTCCTTGGCTTCTTTCCATCTA
CGGCGTGCCCGTGCTCATCCTCAACGCTTTCGTTGTTCTCATCACATTCCTCCAGCACTCGCATCCTGCACTCCCCCA
CTACAATTCGGACGAGTGGGATTGGCTTCGGGGTGCCCTTGCAACAGTCGACCGAGATTATGGATTTCTGAACGAG
GTTTTTCACGATATCACGGATACCCACGTTATTCATCACCTTTTCCCCACCATGCCCCATTACAACGCTAAGGAGGCT
ACTGTGTCGATCCGGCCCATCCTGAAAGATTACTATAAGTTTGATCGTACACCCATTTGGAGAGCTCTCTGGCGGGA
GGCCAAAGAATGTCTTTACGTCGAAGCCGATGGTACCGGCTCGAAGGGAGTCCTCTGGTTTAAATCTAAATTTTAAT
CTAGTGTCTGTGGTATCTAAGCTATTTATCACTCTTTACAACTTCTACCTCAACTATCTACTTTAATAAATGAATATCG
TTTATTCTCTATGATTACTGTATATGCGTTCCTCTAAGACAGAGTCATGTGTAACACTCGCTCTGGAGAGTTAGTCAT
CCGACAGGGTAACTCTAATCTCCCAACACCTTATTAACTCTGCGTAACTGTAACTCTTCTTGCCACGTCGATCTTACT
CAATTTTCCTGCTCATCATCTGCTGGATTGTTGTCTATCGTCTGGCTCTAATACATTTATTGTTTATTGCCCAAACAAC
TTTCATTGCACGTAAGTGAATTGTTTTATAACAGCGTTCGCCAATTGCTGCGCCATCGTCGTCCGGCTGTCCTACCGT
TAGGGGTAGTGTGTCTCACACTACCGAGGTTACTAGAGTTGGGAAAGCGATACTGCCTCGGACACACCACCTGGGT
CTTACGACTGCAGAGAGAATCGGCGTTACCTCTCTCACAAAGCCCTTCAGTGCGGCCGC

FADX gene	*TkFADX*

Promoter	*pTEF*
E. coli strain	JME5215
Y. lipolytica strain	JMY8385

GCGGCCGCTGTCGGGAACCGCGTTCAGGTGGAACAGGACCACCTCCCTTGCACTTCTTGGTATATCAGTATAGGCTG
ATGTATTCATAGTGGGTTTTTCATAATAAATTTACTAACGGCAGGCAACATTCACTCGGCTTAAACGCAAAACGGA
CCGTCTTGATATCTTCTGACGCATTGACCACCGAGAAATAGTGTTAGTTACCGGGTGAGTTATTGTTCTTCTACACAG
GCGACGCCCATCGTCTAGAGTTGATGTACTAACTCAGATTTCACTACCTACCCTATCCCTGGTACGCACAAAGCACT
TTGCTAGAAGGTATAACTTCGTATAGCATACATTATACGAAGTTATTCTGAATTCCGAGAAACACAACAACATGCCC
CATTGGACAGACCATGCGGATACACAGGTTGTGCAGTACCATACATACTCGATCAGACAGGTCGTCTGACCATCAT
ACAAGCTGAACAGCGCTCCATACTTGCACGCTCTCTATATACACAGTTAAATTACATATCCATAGTCTAACCTCTAA
CAGTTAATCTTCTGGTAAGCCTCCCAGCCAGCCTTCTGGTATCGCTTGGCCTCCTCAATAGGATCTCGGTTCTGGCCG
TACAGACCTCGGCCGACAATTATGATATCCGTTCCGGTAGACATGACATCCTCAACAGTTCGGTACTGCTGTCCGAG
AGCGTCTCCCTTGTCGTCAAGACCCACCCCGGGGGTCAGAATAAGCCAGTCCTCAGAGTCGCCCTTAGGTCGGTTCT
GGGCAATGAAGCCAACCACAAACTCGGGGTCGGATCGGGCAAGCTCAATGGTCTGCTTGGAGTACTCGCCAGTGGC
CAGAGAGCCCTTGCAAGACAGCTCGGCCAGCATGAGCAGACCTCTGGCCAGCTTCTCGTTGGGAGAGGGGACTAG
GAACTCCTTGTACTGGGAGTTCTCGTAGTCAGAGACGTCCTCCTTCTTCTGTTCAGAGACAGTTTCCTCGGCACCAGC
TCGCAGGCCAGCAATGATTCCGGTTCCGGGTACACCGTGGGCGTTGGTGATATCGGACCACTCGGCGATTCGGTGAC
ACCGGTACTGGTGCTTGACAGTGTTGCCAATATCTGCGAACTTTCTGTCCTCGAACAGGAAGAAACCGTGCTTAAGA
GCAAGTTCCTTGAGGGGGAGCACAGTGCCGGCGTAGGTGAAGTCGTCAATGATGTCGATATGGGTCTTGATCATGC
ACACATAAGGTCCGACCTTATCGGCAAGCTCAATGAGCTCCTTGGTGGTGGTAACATCCAGAGAAGCACACAGGTT
GGTTTTCTTGGCTGCCACGAGCTTGAGCACTCGAGCGGCAAAGGCGGACTTGTGGACGTTAGCTCGAGCTTCGTAGG
AGGGCATTTTGGTGGTGAAGAGGAGACTGAAATAAATTTAGTCTGCAGAACTTTTTATCGGAACCTTATCTGGGGCA
GTGAAGTATATGTTATGGTAATAGTTACGAGTTAGTTGAACTTATAGATAGACTGGACTATACGGCTATCGGTCCAA
ATTAGAAAGAACGTCAATGGCTCTCTGGGCGCGAATTCGTATAACTTCGTATAGCAGGAGTTATCCGAAGCGAACG
GGGGTTGGCGGCGTATTTGTGTCCCAAAAAACAGCCCCAATTGCCCCAATTGACCCCAAATTGACCCAGTAGCGGG
CCCAACCCCGGCGAGAGCCCCCTTCACCCCACATATCAAACCTCCCCCGGTTCCCACACTTGCCGTTAAGGGCGTA
GGGTACTGCAGTCTGGAATCTACGCTTGTTCAGACTTTGTACTAGTTTCTTTGTCTGGCCATCCGGGTAACCCATGCC
GGACGCAAAATAGACTACTGAAAATTTTTTTGCTTTGTGGTTGGGACTTTAGCCAAGGGTATAAAAGACCACCGTCC
CCGAATTACCTTTCCTCTTCTTTTCTCTCTCTCCTTGTCAACTCACACCCGAAGAATGGGAGGAGGAGAAGGAATCG
AGGTTCGTTCGGGTTCTTCGTCGACTAAACTTGCATTCGGCGAGCGTATCACTCACGCCAAGCCCCCCTTCTCCATTT
CGCAGATCAAAAAGGCAATCCCCCCTCATTGCTTTCAGCGATCTCTCTACCGTAGCTTTAGCTATGTTATTTTCGACT
TCATTTTTGCCTCCACGTTTTATCATATCGCTGCTACTAATTTTCACAGACTCCCTCATCCCCTGCATTATCTCGCATG
GCCTCTTTACTGGTTCTGTCAAGGTTCCGTGTTCACAGGTCTGTGGGTGATTGCCCACGAGTGCGGACATCGAGCCTT
CAGCGACTACCAGCTGGTGGATGATGTTGTTGGTTTCCTTCTTCATACTTCGTTCCTGATCCCTTACTTTTCGTTTAAA
ATTTCTCACCGTCGACACCACTCCAATACAGCCAGCCTTGAACGTGATGAAGTGTTTGTTCCCAAGCCTAAAGCCAA
GATGCCCTGGTATTTTAAACACCTCACGAACCCTCCTGCAAGAGTTCTTATCATTTTCATTACTCTGACCCTGGGATG
GCCTATGTACCTCGCTTTCAATATTTCCGGACGTTTCTACGAACGGTTCACGAGCCACTTTGATCCCAATTCGCCTAT
TTTTTCTGAGAATGAGTGGCTTCAGGTGCACATCTCTAATGCCGGAATCGTGGCCGTTTGGTATCTGCTGTACAAGCT
GGCAGCCGCTAAAGGCATTGCTTGGGTTATTCGGATGTATGTTGTCCCTGTCACCATCATGAACGCCTTTGTGGTTCT
TATTACGTCCCTCCAGCATACTCATCCTAGCTTTCCCTACTACGATAGCACTGAGTGGAACTGGCTTCGAGGCAACCT
GGTTACCCTGGACCGAGACTATGGTATCCTTAATAAGGTGTTTCATAACATCACCGATACCCATGTCGTGCACCATC
TCTTTCCTTCGATGCCTCACTACAACGCCATGGAAGCCACACGTGCCGTTAAACAGGTGCTGGGAGAATACTATCAT
TTTGACGGCACTCCCATTTTTAAAGCAGCCTGGCGTGAGTTTCGGGAGTGCATCTATGTGGAGCCCGACAATGATGA
AGGCGCATCGAGCTCCAGCAAAGGCGTTTTTTGGTTTCGTAATAAACTCTAATCTAGTGTCTGTGGTATCTAAGCTAT
TTATCACTCTTTACAACTTCTACCTCAACTATCTACTTTAATAAATGAATATCGTTTATTCTCTATGATTACTGTATATG
CGTTCCTCTAAGACAGAGTCATGTGTAACACTCGCTCTGGAGAGTTAGTCATCCGACAGGGTAACTCTAATCTCCCA
ACACCTTATTAACTCTGCGTAACTGTAACTCTTCTTGCCACGTCGATCTTACTCAATTTTCCTGCTCATCATCTGCTGG
ATTGTTGTCTATCGTCTGGCTCTAATACATTTATTGTTTATTGCCCAAACAACTTTCATTGCACGTAAGTGAATTGTTT
TATAACAGCGTTCGCCAATTGCTGCGCCATCGTCGTCCGGCTGTCCTACCGTTAGGGGTAGTGTGTCTCACACTACCG
AGGTTACTAGAGTTGGGAAAGCGATACTGCCTCGGACACACCACCTGGGTCTTACGACTGCAGAGAGAATCGGCGT
TACCTCTCTCACAAAGCCCTTCAGTGCGGCCGC

FADX gene	*PgFADX*
Promoter	*pEYK1-4AB*
E. coli strain	JME5334
Y. lipolytica strain	JMY8716

GCGGCCGCTGTCGGGAACCGCGTTCAGGTGGAACAGGACCACCTCCCTTGCACTTCTTGGTATATCAGTATAGGCTG
ATGTATTCATAGTGGGTTTTTCATAATAAATTTACTAACGGCAGGCAACATTCACTCGGCTTAAACGCAAAACGGA
CCGTCTTGATATCTTCTGACGCATTGACCACCGAGAAATAGTGTTAGTTACCGGGTGAGTTATTGTTCTTCTACACAG

GCGACGCCCATCGTCTAGAGTTGATGTACTAACTCAGATTTCACTACCTACCCTATCCCTGGTACGCACAAAGCACT
TTGCTAGAAGGTATAACTTCGTATAGCATACATTATACGAAGTTATTCTGAATTCCGAGAAACACAACAACATGCCC
CATTGGACAGACCATGCGGATACACAGGTTGTGCAGTACCATACATACTCGATCAGACAGGTCGTCTGACCATCAT
ACAAGCTGAACAGCGCTCCATACTTGCACGCTCTCTATATACACAGTTAAATTACATATCCATAGTCTAACCTCTAA
CAGTTAATCTTCTGGTAAGCCTCCCAGCCAGCCTTCTGGTATCGCTTGGCCTCCTCAATAGGATCTCGGTTCTGGCCG
TACAGACCTCGGCCGACAATTATGATATCCGTTCCGGTAGACATGACATCCTCAACAGTTCGGTACTGCTGTCCGAG
AGCGTCTCCCTTGTCGTCAAGACCCACCCCGGGGGTCAGAATAAGCCAGTCCTCAGAGTCGCCCTTAGGTCGGTTCT
GGGCAATGAAGCCAACCACAAACTCGGGGTCGGATCGGGCAAGCTCAATGGTCTGCTTGGAGTACTCGCCAGTGGC
CAGAGAGCCCTTGCAAGACAGCTCGGCCAGCATGAGCAGACCTCTGGCCAGCTTCTCGTTGGGAGAGGGGACTAG
GAACTCCTTGTACTGGGAGTTCTCGTAGTCAGAGACGTCCTCCTTCTTCTGTTCAGAGACAGTTTCCTCGGCACCAGC
TCGCAGGCCAGCAATGATTCCGGTTCCGGGTACACCGTGGGCGTTGGTGATATCGGACCACTCGGCGATTCGGTGAC
ACCGGTACTGGTGCTTGACAGTGTTGCCAATATCTGCGAACTTTCTGTCCTCGAACAGGAAGAAACCGTGCTTAAGA
GCAAGTTCCTTGAGGGGGAGCACAGTGCCGGCGTAGGTGAAGTCGTCAATGATGTCGATATGGGTCTTGATCATGC
ACACATAAGGTCCGACCTTATCGGCAAGCTCAATGAGCTCCTTGGTGGTGGTAACATCCAGAGAAGCACACAGGTT
GGTTTTCTTGGCTGCCACGAGCTTGAGCACTCGAGCGGCAAAGGCGGACTTGTGGACGTTAGCTCGAGCTTCGTAGG
AGGGCATTTTGGTGGTGAAGAGGAGACTGAAATAAATTTAGTCTGCAGAACTTTTTATCGGAACCTTATCTGGGGCA
GTGAAGTATATGTTATGGTAATAGTTACGAGTTAGTTGAACTTATAGATAGACTGGACTATACGGCTATCGGTCCAA
ATTAGAAAGAACGTCAATGGCTCTCTGGGCGCGAATTCGTATAACTTCGTATAGCAGGAGTTATCCGAAGCGAACG
GCGATACGCGTATCGATGCATCTACTTTTCTCTATACTGTACGTTTCAATCTGGGGAAGCGGAATCCCAAAAGGGAA
AGCCGCCGCATTAAGCTCCACAGCGGGAAGCGGAATCCCAAAAGGGAAAGCCGCCGCATTAAGCTCCACAGCGGG
AAGCGGAATCCCAAAAGGGAAAGCCGCCGCATTAAGCTCCACAGCGGGAAGCGGAATCCCAAAAGGGAAAGCCG
CCGCATTAAGCTCCACAGCCTTGCATAATCCGATGACCTGACTAGTGCGGACAAAGACTATTATTTCGAGGCAAGG
CCACCACGTACCGCGGTCCCAAACTTTTGCAAAGCTGAAAACAGCGTGGGGGTCAACGTGGATCAGAAAGAGGGG
CAGATCAGCTTCTATAAGAAGCTCCTTTCCCCACAATTGGCCCACACGACACTTCTACACACTTACACATCTACTGG
ATCCAATGGGCGCAGACGGTACTATGAGCCCCGTGCTTACGAAGCGACGACCTGATCAGGAAATTAATAAGCTCGA
TATTAAACCCAACCACGAGGTCGATATTGCACGACGAGCACCCCACTCTAAGCCCCCTTTTACTCTCAGCGATCTTC
GTTCTGCTATTCCTCCCCACTGTTTCCACCGTTCCCTCCTTATGTCCTCCAGCTACCTTATCCGGGATTTTGCTCTTGCA
TTTCTTTTCTACCATTCCGCTGTGACCTATATCCCCCTCCTTCCTAAACCTCTCGCCTGTATGGCTTGGCCCGTGTACTG
GTTTCTCCAGGGATCTAATATGCTCGGAATTTGGGTTATCGCCCACGAATGCGGACATCAGGCCTTTAGCAATTACG
GTTGGGTCAACGACGCAGTCGGTTTCTTTCTGCATACCAGCCTCCTTGTGCCTTATTTCCCTTTCAAATACTCGCACAG
ACGTCACCACTCCAACACTAATAGCGTTGAGCACGATGAGGTTTTTGTTCCTAGACACAAGGACGGCGTCCAATGGT
ATTATCGGTTCTTCAACAATACGCCTGGCCGTGTCCTCACACTGACTCTTACACTCCTGGTTGGATGGCCTTCCTACC
TTGCTTTTAACGCTTCCGGTAGACCTTATGACGGTTTCGCCTCGCATTACAATCCTAATGCCCAAATTTTTAACCTCCG
TGAGCGTTTCTGGGTGCACGTGTCGAACATCGGCATTCTTGCTATCTACTATATCCTGTACCGACTCGCCACTACGAA
GGGCCTTCCTTGGCTTCTTTCCATCTACGGCGTGCCCGTGCTCATCCTCAACGCTTTCGTTGTTCTCATCACATTCCTC
CAGCACTCGCATCCTGCACTCCCCCACTACAATTCGGACGAGTGGGATTGGCTTCGGGGTGCCCTTGCAACAGTCGA
CCGAGATTATGGATTTCTGAACGAGGTTTTTCACGATATCACGGATACCCACGTTATTCATCACCTTTTCCCCACCAT
GCCCCATTACAACGCTAAGGAGGCTACTGTGTCGATCCGGCCCATCCTGAAAGATTACTATAAGTTTGATCGTACAC
CCATTTGGAGAGCTCTCTGGCGGGAGGCCAAAGAATGTCTTTACGTCGAAGCCGATGGTACCGGCTCGAAGGGAGT
CCTCTGGTTTAAATCTAAATTTTAATCTAGTGTCTGTGGTATCTAAGCTATTTATCACTCTTTACAACTTCTACCTCAA
CTATCTACTTTAATAAATGAATATCGTTTATTCTCTATGATTACTGTATATGCGTTCCTCTAAGACAGAGTCATGTGTA
ACACTCGCTCTGGAGAGTTAGTCATCCGACAGGGTAACTCTAATCTCCCAACACCTTATTAACTCTGCGTAACTGTA
ACTCTTCTTGCCACGTCGATCTTACTCAATTTTCCTGCTCATCATCTGCTGGATTGTTGTCTATCGTCTGGCTCTAATAC
ATTTATTGTTTATTGCCCAAACAACTTTCATTGCACGTAAGTGAATTGTTTTATAACAGCGTTCGCCAATTGCTGCGC
CATCGTCGTCCGGCTGTCCTACCGTTAGGGGTAGTGTGTCTCACACTACCGAGGTTACTAGAGTTGGGAAAGCGATA
CTGCCTCGGACACACCACCTGGGTCTTACGACTGCAGAGAGAATCGGCGTTACCTCTCTCACAAAGCCCTTCAGTGC
GGCCGC

FADX gene	*PgFADX*
Promoter	*pEYD1AB*
E. coli strain	JME5335
Y. lipolytica strain	JMY8717

GCGGCCGCTGTCGGGAACCGCGTTCAGGTGGAACAGGACCACCTCCCTTGCACTTCTTGGTATATCAGTATAGGCTG
ATGTATTCATAGTGGGGTTTTTCATAATAAATTTACTAACGGCAGGCAACATTCACTCGGCTTAAACGCAAAACGGA
CCGTCTTGATATCTTCTGACGCATTGACCACCGAGAAATAGTGTTAGTTACCGGGTGAGTTATTGTTCTTCTACACAG
GCGACGCCCATCGTCTAGAGTTGATGTACTAACTCAGATTTCACTACCTACCCTATCCCTGGTACGCACAAAGCACT
TTGCTAGAAGGTATAACTTCGTATAGCATACATTATACGAAGTTATTCTGAATTCCGAGAAACACAACAACATGCCC
CATTGGACAGACCATGCGGATACACAGGTTGTGCAGTACCATACATACTCGATCAGACAGGTCGTCTGACCATCAT
ACAAGCTGAACAGCGCTCCATACTTGCACGCTCTCTATATACACAGTTAAATTACATATCCATAGTCTAACCTCTAA
CAGTTAATCTTCTGGTAAGCCTCCCAGCCAGCCTTCTGGTATCGCTTGGCCTCCTCAATAGGATCTCGGTTCTGGCCG
TACAGACCTCGGCCGACAATTATGATATCCGTTCCGGTAGACATGACATCCTCAACAGTTCGGTACTGCTGTCCGAG

AGCGTCTCCCTTGTCGTCAAGACCCACCCCGGGGGTCAGAATAAGCCAGTCCTCAGAGTCGCCCTTAGGTCGGTTCT
GGGCAATGAAGCCAACCACAAACTCGGGGTCGGATCGGGCAAGCTCAATGGTCTGCTTGGAGTACTCGCCAGTGGC
CAGAGAGCCCTTGCAAGACAGCTCGGCCAGCATGAGCAGACCTCTGGCCAGCTTCTCGTTGGGAGAGGGGACTAG
GAACTCCTTGTACTGGGAGTTCTCGTAGTCAGAGACGTCCTCCTTCTTCTGTTCAGAGACAGTTTCCTCGGCACCAGC
TCGCAGGCCAGCAATGATTCCGGTTCCGGGTACACCGTGGGCGTTGGTGATATCGGACCACTCGGCGATTCGGTGAC
ACCGGTACTGGTGCTTGACAGTGTTGCCAATATCTGCGAACTTTCTGTCCTCGAACAGGAAGAAACCGTGCTTAAGA
GCAAGTTCCTTGAGGGGGAGCACAGTGCCGGCGTAGGTGAAGTCGTCAATGATGTCGATATGGGTCTTGATCATGC
ACACATAAGGTCCGACCTTATCGGCAAGCTCAATGAGCTCCTTGGTGGTGGTAACATCCAGAGAAGCACACAGGTT
GGTTTTCTTGGCTGCCACGAGCTTGAGCACTCGAGCGGCAAAGGCGGACTTGTGGACGTTAGCTCGAGCTTCGTAGG
AGGGCATTTTGGTGGTGAAGAGGAGACTGAAATAAATTTAGTCTGCAGAACTTTTTATCGGAACCTTATCTGGGGCA
GTGAAGTATATGTTATGGTAATAGTTACGAGTTAGTTGAACTTATAGATAGACTGGACTATACGGCTATCGGTCCAA
ATTAGAAAGAACGTCAATGGCTCTCTGGGCGCGAATTCGTATAACTTCGTATAGCAGGAGTTATCCGAAGCGAACG
GCCCATCGATGGAAACCTTAATAGGAGACTACTTCCGTTTCCTAATTAGGACTTCCGCGACCCCAGACAAAGCGGCT
TGGAGTAGGCCTCGTGTCCGGCCTAGGGCAGAAACAGCTCCGGAACTCGATTGAGAAGCCGTACTCTGGAAAGTCT
AGAGGAAGTTCCAAGGTCGAGTCTCTTCGATATAAAAGGACGCCATGGAAGCTCTGTAGTTCGATATCAAATACTG
ACAACAGTTTCCAAACACACAAACACACACACACACACACACACACATACACAAATGGGCGCAGACGGTACTATG
AGCCCCGTGCTTACGAAGCGACGACCTGATCAGGAAATTAATAAGCTCGATATTAAACCCAACCACGAGGTCGATA
TTGCACGACGAGCACCCCACTCTAAGCCCCCTTTTACTCTCAGCGATCTTCGTTCTGCTATTCCTCCCCACTGTTTCCA
CCGTTCCCTCCTTATGTCCTCCAGCTACCTTATCCGGGATTTTGCTCTTGCATTTCTTTTCTACCATTCCGCTGTGACCT
ATATCCCCCTCCTTCCTAAACCTCTCGCCTGTATGGCTTGGCCCGTGTACTGGTTTCTCCAGGGATCTAATATGCTCG
GAATTTGGGTTATCGCCCACGAATGCGGACATCAGGCCTTTAGCAATTACGGTTGGGTCAACGACGCAGTCGGTTTC
TTTCTGCATACCAGCCTCCTTGTGCCTTATTTCCCTTTCAAATACTCGCACAGACGTCACCACTCCAACACTAATAGC
GTTGAGCACGATGAGGTTTTTGTTCCTAGACACAAGGACGGCGTCCAATGGTATTATCGGTTCTTCAACAATACGCC
TGGCCGTGTCCTCACACTGACTCTTACACTCCTGGTTGGATGGCCTTCCTACCTTGCTTTTAACGCTTCCGGTAGACCT
TATGACGGTTTCGCCTCGCATTACAATCCTAATGCCCAAATTTTTAACCTCCGTGAGCGTTTCTGGGTGCACGTGTCG
AACATCGGCATTCTTGCTATCTACTATATCCTGTACCGACTCGCCACTACGAAGGGCCTTCCTTGGCTTCTTTCCATCT
ACGGCGTGCCCGTGCTCATCCTCAACGCTTTCGTTGTTCTCATCACATTCCTCCAGCACTCGCATCCTGCACTCCCCC
ACTACAATTCGGACGAGTGGGATTGGCTTCGGGGTGCCCTTGCAACAGTCGACCGAGATTATGGATTTCTGAACGA
GGTTTTTCACGATATCACGGATACCCACGTTATTCATCACCTTTTCCCCACCATGCCCCATTACAACGCTAAGGAGGC
TACTGTGTCGATCCGGCCCATCCTGAAAGATTACTATAAGTTTGATCGTACACCCATTTGGAGAGCTCTCTGGCGGG
AGGCCAAAGAATGTCTTTACGTCGAAGCCGATGGTACCGGCTCGAAGGGAGTCCTCTGGTTTAAATCTAAATTTTAA
TCTAGTGTCTGTGGTATCTAAGCTATTTATCACTCTTTACAACTTCTACCTCAACTATCTACTTTAATAAATGAATATC
GTTTATTCTCTATGATTACTGTATATGCGTTCCTCTAAGACAGAGTCATGTGTAACACTCGCTCTGGAGAGTTAGTCA
TCCGACAGGGTAACTCTAATCTCCCAACACCTTATTAACTCTGCGTAACTGTAACTCTTCTTGCCACGTCGATCTTAC
TCAATTTTCCTGCTCATCATCTGCTGGATTGTTGTCTATCGTCTGGCTCTAATACATTTATTGTTTATTGCCCAAACAA
CTTTCATTGCACGTAAGTGAATTGTTTTATAACAGCGTTCGCCAATTGCTGCGCCATCGTCGTCCGGCTGTCCTACCG
TTAGGGGTAGTGTGTCTCACACTACCGAGGTTACTAGAGTTGGGAAAGCGATACTGCCTCGGACACACCACCTGGG
TCTTACGACTGCAGAGAGAATCGGCGTTACCTCTCTCACAAAGCCCTTCAGTGCGGCCGC

FADX gene	*PgFADX*
Promoter	*pEYK1-4AB-coreTEF*
E. coli strain	JME5336
Y. lipolytica strain	JMY8721

GCGGCCGCTGTCGGGAACCGCGTTCAGGTGGAACAGGACCACCTCCCTTGCACTTCTTGGTATATCAGTATAGGCTG
ATGTATTCATAGTGGGGTTTTTCATAATAAATTTACTAACGGCAGGCAACATTCACTCGGCTTAAACGCAAAACGGA
CCGTCTTGATATCTTCTGACGCATTGACCACCGAGAAATAGTGTTAGTTACCGGGTGAGTTATTGTTCTTCTACACAG
GCGACGCCCATCGTCTAGAGTTGATGTACTAACTCAGATTTCACTACCTACCCTATCCCTGGTACGCACAAAGCACT
TTGCTAGAAGGTATAACTTCGTATAGCATACATTATACGAAGTTATTCTGAATTCCGAGAAACACAACAACATGCCC
CATTGGACAGACCATGCGGATACACAGGTTGTGCAGTACCATACATACTCGATCAGACAGGTCGTCTGACCATCAT
ACAAGCTGAACAGCGCTCCATACTTGCACGCTCTCTATATACACAGTTAAATTACATATCCATAGTCTAACCTCTAA
CAGTTAATCTTCTGGTAAGCCTCCCAGCCAGCCTTCTGGTATCGCTTGGCCTCCTCAATAGGATCTCGGTTCTGGCCG
TACAGACCTCGGCCGACAATTATGATATCCGTTCCGGTAGACATGACATCCTCAACAGTTCGGTACTGCTGTCCGAG
AGCGTCTCCCTTGTCGTCAAGACCCACCCCGGGGGTCAGAATAAGCCAGTCCTCAGAGTCGCCCTTAGGTCGGTTCT
GGGCAATGAAGCCAACCACAAACTCGGGGTCGGATCGGGCAAGCTCAATGGTCTGCTTGGAGTACTCGCCAGTGGC
CAGAGAGCCCTTGCAAGACAGCTCGGCCAGCATGAGCAGACCTCTGGCCAGCTTCTCGTTGGGAGAGGGGACTAG
GAACTCCTTGTACTGGGAGTTCTCGTAGTCAGAGACGTCCTCCTTCTTCTGTTCAGAGACAGTTTCCTCGGCACCAGC
TCGCAGGCCAGCAATGATTCCGGTTCCGGGTACACCGTGGGCGTTGGTGATATCGGACCACTCGGCGATTCGGTGAC
ACCGGTACTGGTGCTTGACAGTGTTGCCAATATCTGCGAACTTTCTGTCCTCGAACAGGAAGAAACCGTGCTTAAGA
GCAAGTTCCTTGAGGGGGAGCACAGTGCCGGCGTAGGTGAAGTCGTCAATGATGTCGATATGGGTCTTGATCATGC
ACACATAAGGTCCGACCTTATCGGCAAGCTCAATGAGCTCCTTGGTGGTGGTAACATCCAGAGAAGCACACAGGTT
GGTTTTCTTGGCTGCCACGAGCTTGAGCACTCGAGCGGCAAAGGCGGACTTGTGGACGTTAGCTCGAGCTTCGTAGG

AGGGCATTTTGGTGGTGAAGAGGAGACTGAAATAAATTTAGTCTGCAGAACTTTTTATCGGAACCTTATCTGGGGCA
GTGAAGTATATGTTATGGTAATAGTTACGAGTTAGTTGAACTTATAGATAGACTGGACTATACGGCTATCGGTCCAA
ATTAGAAAGAACGTCAATGGCTCTCTGGGCGCGAATTCGTATAACTTCGTATAGCAGGAGTTATCCGAAGCGAACG
GCGATACGCGTATCGATGCATCTACTTTTCTCTATACTGTACGTTTCAATCTGGGGAAGCGGAATCCCAAAAGGGAA
AGCCGCCGCATTAAGCTCCACAGCGGGAAGCGGAATCCCAAAAGGGAAAGCCGCCGCATTAAGCTCCACAGCGGG
AAGCGGAATCCCAAAAGGGAAAGCCGCCGCATTAAGCTCCACAGCGGGAAGCGGAATCCCAAAAGGGAAAGCCG
CCGCATTAAGCTCCACAGCCTTGCATAATCCGATGACCTGACTAGTGCGGTTGGGACTTTAGCCAAGGGTATAAAA
GACCACCGTCCCCGAATTACCTTTCCTCTTCTTTTCTCTCTCTCCTTGTCAACTCACACCCGAAGGATCCCACAAATG
GGCGCAGACGGTACTATGAGCCCCGTGCTTACGAAGCGACGACCTGATCAGGAAATTAATAAGCTCGATATTAAAC
CCAACCACGAGGTCGATATTGCACGACGAGCACCCCACTCTAAGCCCCCTTTTACTCTCAGCGATCTTCGTTCTGCT
ATTCCTCCCCACTGTTTCCACCGTTCCCTCCTTATGTCCTCCAGCTACCTTATCCGGGATTTTGCTCTTGCATTTCTTTT
CTACCATTCCGCTGTGACCTATATCCCCCTCCTTCCTAAACCTCTCGCCTGTATGGCTTGGCCCGTGTACTGGTTTCTC
CAGGGATCTAATATGCTCGGAATTTGGGTTATCGCCCACGAATGCGGACATCAGGCCTTTAGCAATTACGGTTGGGT
CAACGACGCAGTCGGTTTCTTTCTGCATACCAGCCTCCTTGTGCCTTATTTCCCTTTCAAATACTCGCACAGACGTCA
CCACTCCAACACTAATAGCGTTGAGCACGATGAGGTTTTTGTTCCTAGACACAAGGACGGCGTCCAATGGTATTATC
GGTTCTTCAACAATACGCCTGGCCGTGTCCTCACACTGACTCTTACACTCCTGGTTGGATGGCCTTCCTACCTTGCTTT
TAACGCTTCCGGTAGACCTTATGACGGTTTCGCCTCGCATTACAATCCTAATGCCCAAATTTTTAACCTCCGTGAGCG
TTTCTGGGTGCACGTGTCGAACATCGGCATTCTTGCTATCTACTATATCCTGTACCGACTCGCCACTACGAAGGGCCT
TCCTTGGCTTCTTTCCATCTACGGCGTGCCCGTGCTCATCCTCAACGCTTTCGTTGTTCTCATCACATTCCTCCAGCAC
TCGCATCCTGCACTCCCCCACTACAATTCGGACGAGTGGGATTGGCTTCGGGGTGCCCTTGCAACAGTCGACCGAGA
TTATGGATTTCTGAACGAGGTTTTTCACGATATCACGGATACCCACGTTATTCATCACCTTTTCCCCACCATGCCCCA
TTACAACGCTAAGGAGGCTACTGTGTCGATCCGGCCCATCCTGAAAGATTACTATAAGTTTGATCGTACACCCATTT
GGAGAGCTCTCTGGCGGGAGGCCAAAGAATGTCTTTACGTCGAAGCCGATGGTACCGGCTCGAAGGGAGTCCTCTG
GTTTAAATCTAAATTTTAATCTAGTGTCTGTGGTATCTAAGCTATTTATCACTCTTTACAACTTCTACCTCAACTATCT
ACTTTAATAAATGAATATCGTTTATTCTCTATGATTACTGTATATGCGTTCCTCTAAGACAGAGTCATGTGTAACACT
CGCTCTGGAGAGTTAGTCATCCGACAGGGTAACTCTAATCTCCCAACACCTTATTAACTCTGCGTAACTGTAACTCTT
CTTGCCACGTCGATCTTACTCAATTTTCCTGCTCATCATCTGCTGGATTGTTGTCTATCGTCTGGCTCTAATACATTTAT
TGTTTATTGCCCAAACAACTTTCATTGCACGTAAGTGAATTGTTTTATAACAGCGTTCGCCAATTGCTGCGCCATCGT
CGTCCGGCTGTCCTACCGTTAGGGGTAGTGTGTCTCACACTACCGAGGTTACTAGAGTTGGGAAAGCGATACTGCCT
CGGACACACCACCTGGGTCTTACGACTGCAGAGAGAATCGGCGTTACCTCTCTCACAAAGCCCTTCAGTGCGGCCG
C

Supplementary Table S4. Primers used in the study.

Name	Sequence (5′ – 3′)
**pTEF-internal-Fw**	TCTGGAATCTACGCTTGTTCAG
**pEYK-internal-Fw**	GTACGTTTCAATCTGGGGAAGCGG
**pEYD-internal-Fw**	CCCATCGATGGAAACCTTAATAGGAGACTACTTCC
**PgFADX-Rev**	CCAGAGGACTCCCTTCGAGCC
**TkFADX-Rev**	CGCCTTTGCTGGAGCTCGATGC
**pTEF-internal-Fw2**	CCAATTGACCCCAAATTGACC
**DGA2-Rev**	ATGAGGCGAGTTTGAGAGACAG
